# The Trajectory of Targets and Critical Lures in the Deese/Roediger–McDermott Paradigm: A Systematic Review

**DOI:** 10.3389/fpsyg.2021.718818

**Published:** 2021-12-03

**Authors:** Patricia I. Coburn, Kirandeep K. Dogra, Iarenjit K. Rai, Daniel M. Bernstein

**Affiliations:** ^1^Department of Psychology, Kwantlen Polytechnic University, Surrey, BC, Canada; ^2^Simon Fraser University, Burnaby, BC, Canada

**Keywords:** Deese/Roediger–McDermott (DRM) paradigm, critical lures, targets, trajectory, false memory, Fuzzy Trace Theory, delay, associative-activation theory

## Abstract

The Deese/Roediger–McDermott (DRM) paradigm has been used extensively to examine false memory. During the study session, participants learn lists of semantically related items (e.g., pillow, blanket, tired, bed), referred to as targets. Critical lures are items which are also associated with the lists but are intentionally omitted from study (e.g., sleep). At test, when asked to remember targets, participants often report false memories for critical lures. Findings from experiments using the DRM show the ease with which false memories develop in the absence of suggestion or misinformation. Given this, it is important to examine factors which influence the generalizability of the findings. One important factor is the persistence of false memory, or how long false memories last. Therefore, we conducted a systemic review to answer this research question: What is the persistence of false memory for specific items in the DRM paradigm? To help answer this question our review had two research objectives: (1) to examine the trajectory of target memory and false memory for critical lures and (2) to examine whether memory for targets exceeded false memory for critical lures. We included empirical articles which tested memory for the same DRM lists with at least two testing sessions. We discuss the results with respect to single-session delays, long-term memory recall and recognition, remember and know judgments for memory, and the effect of development, valence, warning, and connectivity on the trajectory of memory. Overall, the trajectory of targets showed a relatively consistent pattern of decrease across delay. The trajectory of critical lures was inconsistent. The proportion of targets versus critical lures across delay was also inconsistent. Despite the inconsistencies, we conclude that targets and critical lures have a dissimilar trajectory across delay and that critical lures are more persistent than targets. The findings with respect to long-term recall and recognition are consistent with both Fuzzy Trace Theory and Associative-Activation Theory of the DRM effect. The generation of false memory with brief delays (3–4 s) is better explained by Associative-Activation Theory. Examining the connectivity between target items, and critical lures, and the effect that has during study and retrieval, can provide insight into the persistence of false memory for critical lures.

## Introduction

Imagine that your partner asks you to shop for produce, providing you with a list of fruits to buy. You forget the list, and then buy what you think was on the list. When you return home with your bounty, your partner asks why you bought oranges. You say that you forgot the list, but you remembered that oranges were on it. This, however, is a false memory, because oranges were not on the list. Memory is vulnerable to errors of omission (information that was present initially but not retrieved later), as well as errors of commission (information that was absent initially but was retrieved later). In the latter category, intrusions can arise internally (self-generated; this is the type of error you made when you remembered that oranges were on the list) or externally through post-event information. Internally and externally generated errors fall in the broad category of false memory, although there is evidence that the two may be unrelated and have distinct underlying mechanisms (Ost et al., 2013; Bernstein et al., 2018; Nichols and Loftus, 2019).

One technique that has been used extensively to examine factors associated with false memory is the Deese/Roediger–McDermott (DRM) paradigm (Deese, 1959; Roediger and McDermott, 1995). Findings from research with this paradigm show that false memories develop rapidly and effortlessly (Read, 1996). In a standard DRM paradigm, during the study phase, individuals learn lists of semantically associated words (e.g., grapes, apples, lemons, melon, limes, and strawberries). At test, individuals try to remember the words from the study phase. True memories occur when participants remember the words presented at study – *targets.* Many individuals falsely remember semantically associated words that were absent at study (e.g., oranges). Researchers intentionally omit these associated words from study and refer to these as *critical lures*. Individuals remember critical lures at a higher rate than semantically unrelated words that were also absent at study (e.g., books). The latter are called *distractors* or *foils*. False memory has been tested in the DRM paradigm with different measures of memory, such as recall, recognition, or remember/know judgments. There is an extensive literature on the DRM which illustrates the importance of semantic encoding in memory. The findings also show that individuals form false memories in the absence of external suggestion or post-event information.

The ease with which individuals develop false memories in the DRM paradigm has contributed to research on the fallibility of memory, and the implications of false memory in real-world contexts. Therefore, it is important to study factors which influence the generalizability of the effect. One such factor is the persistence of false memory, or how long false memories last. In real-world contexts, an individual will be required to remember information after a delay, sometimes even after a lengthy delay. As in the example above, even if you provide the initial report quickly, you may have to provide or remember this information weeks or months later. Providing multiple reports may increase the likelihood that a memory error will occur. This is especially true if misinformation or suggestion is introduced (e.g., Loftus et al., 1978; Belli, 1989). However, the DRM paradigm shows that false memories can occur without suggestion. Furthermore, some research shows that across delay, these false memories remain relatively stable, or even inflate, compared to true memories.

False memories in real-world settings can have serious consequences. Now imagine that 30 min into lunch with a friend, you realize that you have an itchy raised rash on your neck. You are finding it difficult to breathe so you decide to go directly to emergency. There you are quickly greeted by a doctor who gives you an injection of epinephrine. The doctor suggests you write down everything you had at lunch. The task seems relatively straightforward: You had grapes, apples, lemons, melon, limes, strawberries, oranges, and blueberries. However, your memory of eating oranges is false. In this example, falsely remembering an orange at lunch could make you avoid the wrong food in the future, while consuming foods that could induce another potentially fatal reaction.

While it can be argued that the trajectory of true memory (targets) and false memory (critical lures) has practical importance, the trajectory of targets and critical lures in the DRM paradigm also has important theoretical implications. Two dominant theoretical explanations for false memory of critical lures in the DRM are the Associative-Activation Theory (Howe et al., 2009), informed by the activation monitoring theory (Roediger et al., 2001a), and Fuzzy Trace Theory (Reyna and Brainerd, 1995). Associative-Activation Theory suggests that critical lures are activated through spreading activation among pre-existing mental networks (Colins and Loftus, 1975). That is, the activation of a target word during the study phase initiates the activation of other words, including words that were not presented during study.

Alternatively, gist theories suggest that critical lures are generated because individuals extract the underlying meaning associated with the list items; critical lures have high semantic relatedness to the list items (see Gallo, 2010). Fuzzy Trace Theory is one popular gist theory used to explain the trajectory of targets and critical lures in the DRM. According to Fuzzy Trace Theory, information encoded in memory forms two traces: Verbatim and gist (Reyna and Brainerd, 1995; Brainerd et al., 1999). The verbatim trace contains item-specific information, while the gist contains mainly underlying meaning of the information without perceptual details (Brainerd et al., 2006). Fuzzy Trace Theory suggests that false memories arising in the DRM paradigm result from representation of the gist that occurs during encoding of the semantic associates on the lists. Gist memory tends to decay more slowly than verbatim memory (Kintsch et al., 1990; Reyna and Kiernan, 1994, 1995). Hence, if false memory for critical lures is due to gist formation, memory for critical lures and targets should have distinct trajectories. Based on Fuzzy Trace Theory, target memory should decrease more quickly than critical lure memory across delay.

Conversely, research that shows similar trajectories between targets and critical lures could indicate that the Associative-Activation Theory better explains false memory of critical lures. The Associative-Activation theory argues that critical lures are activated during the encoding phase due to the pre-existing associations with the items on DRM lists. The theory generally does not propose differences between targets and critical lures that would result in distinct trajectories across delay. Given the theory proposes that false memories for critical lures arise due to their association with targets, variation in how the items are associated could affect the persistence of targets and critical lures across a delay. This can include the number of items on the study lists, and the ease with which the critical lures are generated in free association from the targets (backward association strength), as well as the speed or automaticity of activation (Otgaar et al., 2019).

We conducted a systematic review of the empirical studies that examined the trajectory of memory across time using the DRM paradigm. We conducted our review to answer this research question: What is the persistence of false memory for a specific item in the DRM paradigm? Our review had two research objectives: (1) to examine the trajectory of target memory and false memory for critical lures and (2) to examine whether target memory exceeds false memory for critical lures. The answers to these research questions have practical and theoretical importance. Firstly, given that false memories that are generated in lab-based experiments are often generalized to real-world contexts, understanding how long false memories can occur after the encoding phase is critical to understanding the consequences of false memories (Bernstein and Loftus, 2009). Secondly, understanding the trajectory of targets and critical lures provides insight into the theoretical explanations of false memory. Experimental factors may influence the trajectory of memory in the DRM paradigm, including how researchers measure memory, the number of lists they use, the number of items per list, whether they manipulated delay within or between subject, and whether they included an immediate recall condition. We discuss the results with respect to the trajectory of memory (both true and false) from short to long-term, the trajectory of recall and recognition memory across long delays, the trajectory of remember/know judgments, the trajectory of memory in children and youth, and the effect of connectivity on the trajectory of memory.

## Method

Two co-authors on this review (KD and IR) conducted independent searches in Google Scholar with the key terms, “Delay” AND “Deese-Roediger-McDermott.” KD searched for papers published between 1970 and 2010 and conducted this search between January 6 and January 17, 2021. IR searched for papers published between 2011 and 2021 and conducted this search between January and March 2021. The initial search yielded 1109 hits. KD and IR examined titles and abstracts to determine whether articles met our inclusion criteria: Empirical articles using the DRM paradigm with delay as a manipulated variable (either between or within subject). The first author (PC) developed these inclusionary criteria before the search started; so, we set out to answer this central question. Experiments had to have a minimum of two testing sessions with the same dependent variable at both sessions (i.e., recall followed by recall; or recognition followed by recognition), and the same study lists needed to be used during the testing sessions. We excluded experiments that had additional study sessions at subsequent testing, because we were interested in the trajectory of memory without additional encoding. There were some articles which manipulated delay across experiments. We included these in our sample but note the limitation to this methodological approach. Our criteria resulted in 38 articles. Finally, we examined the reference lists of the included articles to ensure relevant articles were not overlooked. The latter resulted in two additional articles (*N* = 40). All authors met bi-weekly between December 2020 and May 2021 to discuss the criteria and ensure every paper met the inclusion criteria. If there was any confusion whether an article should be included, all authors read the article, and everyone discussed how it fit with the inclusion criteria. Our Results and Discussion focus on targets and critical lures; we only discuss distractors when relevant to interpreting the results.

## Results

Summaries of methods and results for all included articles appear in Supplementary Materials Table 1.

### Single-Session Delay

The trajectory of memories in the DRM paradigm can yield different patterns. Memories can decrease, remain stable, or increase across delay. This can apply to true and false memories independently or in tandem. We refer to these patterns as decrease, stability, and inflation. Regardless of which trajectory true and false memories follow, the proportion of true memories compared to false memories can also vary. Individuals may report proportionally more true than false memories, more false than true memories, or equal numbers of true and false memories.

Perhaps unsurprisingly, the trajectory of false memory depends on the length of delay being manipulated. More surprising to readers unfamiliar with the DRM is that false memories can occur using this paradigm in as little as 3–4 s. And while false memories have been consistently observed with brief delays (McDermott, 1996; Flegal et al., 2010; Festini and Reuter-Lorenz, 2013), the trajectory of true and false memories is inconsistent. For example, in one study, participants viewed 4-item lists of semantically related words and were then probed with a single word (target, unrelated distractor, or critical lure) after a short-term 3–4 s delay or on a surprise long-term recognition test occurring approximately 20 min later (Flegal et al., 2010). The results showed that target recognition decreased from the short-term to the long-term tests while critical lure recognition remained stable across these tests. Despite this, target recognition remained higher than critical lure recognition across delay. Other studies have shown delayed inflation of critical lures with short delays (8–20 min). For example, Festini and Reuter-Lorenz (2013) observed delayed inflation of critical lure recognition across an 8-min delay. As well, Olszewska et al. (2015) observed inflation of critical lure recognition from short-term (3–4 s delay) to long-term (20-min delay) memory. This shows that delayed inflation of critical lures can occur between short and long-term memory.

In many of the studies examining target and critical lure memory within brief periods, participants viewed short lists of words and then completed recognition tests. Recognition tests sometimes yield higher rates of critical lures compared to recall tests (e.g., McEvoy et al., 1999; Stadler et al., 1999). Importantly, the decrease in target recognition and stability of critical lure recognition across a brief delay has also been shown in recall. For example, McDermott (1996) had participants listen to 24 lists. Following each list, participants either recalled the words immediately or after a 30-s delay. Target recall was higher than false recall of critical lures at immediate recall. However, target recall decreased from immediate to delayed recall, while critical lure recall remained stable. Moreover, after the 30-s delay, rates of target and critical lure recall were equivalent. These results are consistent with McEvoy et al. (1999; Experiment 3) who observed lower target recall and stable critical lure recall after a 1-min delay.

The studies described thus far show that false memories for critical lures develop rapidly. This could support gist, or semantic encoding even in short-term memory. These findings are somewhat surprising given that at short delays, memory for the original lists should be strong and permit individuals to recognize memory errors of commission. Recall and recognition of critical lures in such brief periods could support the Associative-Activation Theory of false memory.

Despite the inconsistency and range of methodologies used to examine the trajectory of memory with brief delays, the results are relatively consistent: Target memory begins to decline rapidly, while critical lure memory remains stable, and possibly increases with delays of 20 min or less. The dissimilar trajectory of targets and critical lures across a 20-min delay is consistent with Fuzzy Trace Theory, however, it is arguably also consistent with Association-Activation Theory. Repeated activation of a critical lure in memory, due to multiple associated targets being presented during study, could result in a stronger representation of the critical lure than targets after a delay. This could depend on the association strength between the targets and critical lure.

### Long-Term Recall

When examining the trajectory of targets and critical lures in long-term memory, some research shows that target recall declines more rapidly than false recall of critical lures (Payne et al., 1996; Brainerd et al., 2003; Sherman and Kennerley, 2014; although see Pardilla-Delgado and Payne, 2017). For example, Brainerd et al. (2003) observed delayed inflation of critical lures but not targets over three testing sessions (2-min filler, 5-min test/session). Additionally, participants recalled critical lures at higher rates than targets across all testing sessions. Brainerd et al. (2003) proposed that repeated attempts at recalling semantically related lists provides an opportunity to practice the gist recall processes. With no further opportunity to study the lists, there would be no expectation to strengthen memory for the targets; target memory or true memory relies on access to the verbatim traces rather than constructive processes associated with the gist memory processes (Brainerd et al., 2003). Alternatively, providing an opportunity to access the verbatim traces increases target memory. In a follow-up experiment, increasing the study sessions from one to three, when paired with increased testing, yielded higher rates of target memory than critical lure memory (Brainerd et al., 2003). One might expect, however, that inflation should emerge on targets as well as critical lures across three tests in a single session (21-min) because of the testing effect. Research on the testing effect shows that retrieving information (e.g., through testing) improves memory on subsequent tests (e.g., Roediger and Butler, 2011). Indeed, the testing effect has been shown to be relevant for target memory in the DRM. For example, McDermott (1996) reported that recall of targets and critical lures was higher when there was a previous testing session compared to when there was no previous testing session. However, even though the testing effect emerged, target recall and false recall of critical lures decreased across a 2-day delay. This decrease was smaller for targets and critical lures if participants had previously completed a recall test than if they had not (McDermott, 1996).

Critical lure recall has been shown to be more stable than target recall across even longer delays. For example, Toglia et al. (1999) presented participants with five auditory lists of semantically related words and instructed them to recall either immediately, 1 week, or 3 weeks later. Across the retention intervals, target recall decreased while critical lure recall remained stable. Brainerd et al. (2003) also reported that target recall decreased over a 1-week delay. These findings differ from those of Thapar and McDermott (2001, Experiment 1) who administered a surprise recall task either immediately, 2 days, or 7 days later. But while the decrease from immediate test to day 2 was steeper for target recall than critical lure recall, both decreased. There was little evidence of stability or delayed inflation of critical lures from immediate to a 7-day delay.

Work by Seamon et al. (2002) allows for the trajectory of memory to be examined beyond 1 week. Participants recalled either at a 2-week delay or a 2-month delay. In the delay from 2-weeks to 2-months, critical lure recall decreased while target recall did not. This finding suggests that although false recall of critical lures may be more stable than recall of targets across delays up to 2 weeks, lengthier delays (over 2 weeks) will cause steeper decreases for the critical lures. Even with the steeper decrease for critical lures from 2 weeks to 2 months, critical lures remained higher than targets at the 2-week and 2-month timepoints.

Recall of associated theme items has also been extended to applied settings. For example, Sherman and Kennerley (2014) presented participants with songs from popular artists. Participants recalled the songs 5 min and 1 week after study. During Test 1, participants recalled more target songs than critical lures (biggest hit by the artist, not presented at study; 5%). Recall of target songs decreased while recall of critical lures increased across time. This research shows how false memories produced with the DRM paradigm generalize to real-world settings. Moreover, this work shows that false memories may persist to the same degree as, if not more than, true memories for at least a period of 1 week.

There is also evidence that across delay, false memory for critical lures will exceed true memory for targets. For example, McDermott (1996, described above) tested word recall after a 2-day delay. Initially, participants had either recalled immediately or after completing arithmetic problems for 30 s. After the 2-day delay, critical lure recall exceeded target recall, even though both decreased from the short to long delay. The propensity for critical lure recall to be higher than target recall was also observed in research using a 1- and 2-day delay. In this study, individuals falsely recalled proportionally more critical lures than targets at both time periods (Pardilla-Delgado and Payne, 2017). False recall of critical lures has been shown to exceed target recall across 1-week delays (Thapar and McDermott, 2001; Sherman and Kennerley, 2014) and even across a 2-month delay (Seamon et al., 2002).

Overall, these results show a decline of target recall and stability or delayed inflation of critical lure recall, even after a 3-week delay (Toglia et al., 1999; Brainerd et al., 2003; Sherman and Kennerley, 2014). This stability of critical lure recall also occurs with confidence ratings; target recall confidence declines across delay, but critical lure recall confidence is unaffected by delay (Toglia et al., 1999). While much of the research with recall shows a steeper decrease for targets than critical lures across delay, the findings are mixed with respect to the stability or inflation of critical lures across delay (Thapar and McDermott, 2001, Experiment 1). Importantly there is also evidence that despite target recall being higher than critical lure recall when tested immediately after study, critical lure recall is higher than target recall across delays from 1 day up to 2 months. Thapar and McDermott (2001, Experiment 1) observed that target recall decreased more rapidly than critical lure recall over a 2-day delay. However, there was little evidence of stability or delayed inflation of critical lures as Fuzzy Trace Theory would predict (Thapar and McDermott, 2001, Experiment 1). Fuzzy Trace Theory predicts that critical lures would remain stable or increase across delays because they rely on gist. Failure to observe persistence of critical lures in Thapar and McDermott could be due to how they manipulated delay, or how they presented the stimuli.

In sum, participants tend to show decreased recall of targets and relatively stable or inflated memory for critical lures with delays up to 3 weeks. There is also evidence that despite target recall being higher than critical lure recall when tested immediately after study, critical lure recall is higher than target recall across delays from 1 day to 2 months. These findings are consistent with Fuzzy Trace Theory which predicts that the memory for gist will be stronger across a delay as the verbatim trace becomes less accessible. The results could also be consistent with Associative-Activation Theory. In instances where there is a strong association between the targets and the critical lures, repeated activation of a critical lure through presentation of multiple targets could result in a stronger memory representation for the critical lure than an individual target, across a delay.

### Long-Term Recognition

Deese/Roediger–McDermott recognition findings are less consistent than DRM recall findings. Some studies have shown that target recognition decreases more rapidly than critical lure recognition across long delays (Brainerd et al., 1995, Experiment 2; Payne et al., 1996; Thapar and McDermott, 2001; Parker et al., 2019; Houben et al., 2020, Experiment 1). Other studies have not shown this effect (Lampinen and Schwartz, 2000; Neuschatz et al., 2001; Wang et al., 2017).

For example, Houben et al. (2020) compared two time points (immediate versus 48-h delay) in two experiments. Participants learned five neutral and five negative 10-word DRM lists. Target recognition was higher at Time 1 than Time 2 but there was no effect of delay on critical lure recognition. The decrease in target recognition across a 2-day delay is consistent with Ebbinghaus’s (1913) forgetting curve (see also Lampinen et al., 2005); however, the stable critical lure recognition over delay in Houben et al. is inconsistent with Lampinen et al. (2005) who observed an increase in critical lure recognition across delay. Another study by Lampinen and Schwartz (2000) showed that target recognition and critical lure recognition declined at a similar rate across a 48-h delay. In this study, participants listened to six lists before completing a 48-word recognition test immediately or 2 days afterward. Results showed a decrease in target recognition and corrected critical lure recognition across delay. Across two experiments, target recognition declined more than critical lure recognition for only non-corrected critical lures in Experiment 1. This pattern did not hold for corrected critical lure recognition in Experiment 1 or corrected or uncorrected critical lure recognition in Experiment 2 (Lampinen and Schwartz, 2000). Corrected critical lure scores address response bias by considering responding for non-related and related non-presented words. Non-corrected critical lure recognition does not account for response bias; it simply examines overall critical lure recognition. Bias-corrected scores are commonly reported when participants complete recognition tests (e.g., Brainerd and Reyna, 2018).

Whether recall is present during study likely influences the stability of false recognition across time. Importantly this could also influence the pattern of target recognition. In work by Payne et al. (1996), participants studied 16 lists. Immediately following each list, participants either recalled all the words or they completed arithmetic problems. After presentation of all the lists, half the participants completed a 384-item recognition test while the other participants returned 24-h later to complete the recognition test. Although target recognition decreased across delay, critical lure recognition remained stable. Participants were also more likely to report that a word was old (present at study) if the word belonged to a list which involved immediate recall rather than arithmetic at study. This was true for both types of recognition, but the effect was larger for target than critical lure recognition.

There is evidence that individuals will reject a critical lure in a recognition task if they can remember a specific target (tired) on the list contrary to the lure (sleepy) – *recollection rejection*. The idea of recollection rejection is consistent with Fuzzy Trace Theory, where participants reject a critical lure because they have access to a verbatim trace. In a study conducted by Lampinen et al. (2005), participants performed a think-aloud task during the study and test phase to examine what strategies participants used to reject false memories. Recollection rejection occurred when participants rejected a word (e.g., sleep) because they remembered a different word being present (e.g., tired). Alternatively, *distinctiveness* occurred when the presence of that word should have evoked a specific memory (e.g., I would have remembered the word, needle, because I hate needles; Lampinen et al., 2005). The recognition test occurred either immediately or 48 h after the study phase and included targets, critical lures, non-presented associates that had a weaker association strength than the critical lures, and distractors from the non-studied lists. For all distractors and non-presented associates, distinctiveness was the most common strategy; however, for critical lures, recollection rejection was the most common strategy. While the use of both strategies decreased across delay, recollection rejection remained the most common strategy for critical lures.

A less frequent approach to examining the trajectory of true and false memories is to use a lexical decision task. Studies using reaction time on lexical decision tasks with DRM word lists yield mixed results. McKone (2004) had participants complete an intervening lexical decision task between a study phase and a recognition phase that occurred 3 or 10 min afterward. The percent of targets and critical lures was similar and there were no differences in delay. On the lexical decision task, targets were identified more quickly than targets from unstudied lists, but the reaction time for critical lures and lures from non-presented lists was the same. This pattern occurred across delay. McKone argued that the lexical decision task could distinguish between targets and critical lures across delay. These findings are inconsistent with Sergi et al. (2014), where participants completed a lexical decision task for targets, non-word targets, critical lures, new non-words, and unrelated new words. At an immediate test, a 3-min delay, and a 10-min delay, reaction time for targets and critical lures was shorter than the other categories and there were no differences in reaction time between targets and critical lures. Sergi and colleagues argued that the activation levels of targets and critical lures were similar, and the observed increase in reaction time for the lexical decision task was the same for both after a 10-min delay. The authors argued for an activation theory of false memory formation in the DRM. This argument is supported by the observation of latency scores on the lexical decision task being equivalent for targets and critical lures.

There is some evidence that across a delay, critical lure recognition will be equivalent (Thapar and McDermott, 2001) or higher than target recognition (Brainerd et al., 1995; Huff et al., 2012; Pardilla-Delgado and Payne, 2017). For example, Pardilla-Delgado and Payne (2017) had participants complete the recognition test either 24 h or 48 h after study. There was no effect of delay on target or critical lure recognition, but target recognition was lower than critical lure recognition at both testing points.

In sum, the evidence is mixed for DRM recognition after long delays. Some studies show target recognition decreases more rapidly than critical lure recognition across delay (Brainerd et al., 1995, Experiment 2; Payne et al., 1996; Thapar and McDermott, 2001; Houben et al., 2020). Others have not shown this effect (Lampinen and Schwartz, 2000; Brainerd et al., 2001; Neuschatz et al., 2001; Wang et al., 2017). There is evidence that individuals use recollection rejection when judging critical lures. This strategy is consistent with the Fuzzy Trace Theory prediction that access to the verbatim trace may be used to reject gist-based critical lures. Sergi et al. (2014) used a lexical decision task to argue that targets and critical lures were activated and behaved similarly across delay, a finding more consistent with the Association-Activation Theory than Fuzzy Trace Theory. Finally, there is also some evidence that across delay, critical lure recognition exceeds target recognition. The inconsistency in findings derived from studies using recognition is likely due to the same methodology variations seen in free recall (e.g., between-subjects designs, number of lists, number of items, whether the items were recalled directly after each list).

### Remember/Know Judgments

Theoretical explanations for false memory in the DRM paradigm suggest that the subjective experience of remembering should differ for targets and critical lures (Neuschatz et al., 2001). Based on Fuzzy Trace Theory, perceptual information from the verbatim trace could be integrated with the gist information, especially if the verbatim trace is no longer fully accessible. This could result in the subjective experience of the gist representation (false memory) being rich and resembling that of the verbatim trace. This would be expected to increase with delay due to verbatim decay (see Neuschatz et al., 2001). One way to study the subjective experience of memory is to have participants provide a remember or know judgment for each recognized word (Tulving, 1985; Gardiner and Java, 1990). For example, in Payne et al. (1996) participants provided more remember judgments at Time 1 than Time 2. This decrease in remember judgments occurred for lists followed by arithmetic but not for lists followed by immediate recall.

Neuschatz et al. (2001) found that after 48 h, participants could distinguish true from false items on the DRM using a memory characteristics questionnaire (Johnson et al., 1988). The memory characteristics questionnaire asks participants to rate the experience of remembering an item (e.g., what it sounded like, placement within the list, and what types of reactions the person had when the word was presented). At immediate test, participants provided more remember judgments for studied words than for critical lures, but this difference did not persist after the 48-h delay. However, participants’ responses on the memory characteristics questionnaire differed for studied words and critical lures. That is, participants reported remembering more perceptual information for studied words than critical lures. This suggests that participants can distinguish between targets and critical lures based on some perceptual details for at least 2 days following the study phase. Neuschatz et al. (2001) argued that this is inconsistent with the notion that perceptual details for true memories fade more quickly than those for false or suggested memories, as proposed by Belli and Loftus (1994). It was noted that the findings may not generalize to situations where false memory persists beyond 2 days. It may be that for longer delays, perceptual information for false memory is less vulnerable to decay than that of true memory. For example, to examine false memory in an applied setting, participants watched simulated television programming with advertisements of five associated, but interspersed products (e.g., beers, cars, and banks; Sherman et al., 2015). The researchers observed delayed inflation of critical lures after a 1-week delay. Additionally, remember judgments for target brands and filler items remained stable across time, while remember judgments for critical lures increased across time.

In sum, Fuzzy Trace Theory would predict that the subjective experience of associating rich, perceptual detail with target memory should decrease across delay. As the verbatim trace decays, the subjective experience associated with critical lures should resemble that of targets. The research to date has yet to show this pattern definitively.

### Factors That Influence Persistence of Targets and Critical Lures

We are interested in the persistence of false memory in the DRM paradigm. Up to this point, our review focused on examining the trajectory of targets and critical lures with common measures of memory (recall, recognition, remember/know, and lexical decision tasks). The following sections explore other facets of the DRM that can interact with the effect of delay on targets and critical lures. We feel these facets contribute to the understanding of the trajectories as well as the theoretical explanations of how targets and critical lures persist across delay. For this reason, we included a small overview of: The effects of warning, valence, development, and connectivity on the trajectory of targets and critical lures in the DRM.

### The Effect of Warning on Trajectory of Memories

Some researchers have examined the effect of warning participants about false memories in the DRM paradigm. Generally, individuals learn that the study lists contain items associated with one another. They also learn that during the memory test they will encounter words that are associated with the original study lists but were not presented during study. Participants are asked to avoid recalling or recognizing these words. In many studies examining the effect of warning, participants receive a single study session with or without warning and a single testing session (e.g., McDermott and Roediger, 1998; Gallo et al., 2001). We found only one study that included a warning between Test 1 and Test 2, following a single study session. Miller et al. (2011) had two detailed warning conditions and a no-warning control condition. In the critical lure warning condition, participants heard an explanation of critical lures and learned techniques to prevent false memories. In the criterion warning condition, participants were warned about saying old to any related words. The critical lure warning had no effect on targets or critical lure recognition; however, the criterion warning condition decreased both target and critical lure recognition from Test 1 to Test 2.

In a study by Wang et al. (2017), individuals were told that they falsely recognized a word on Test 1 before they completed Test 2. Participants then completed the compound remote associate task (CRAT). The solutions to the CRAT corresponded to the targets and critical lures from the DRM lists. While being challenged affected CRAT solutions (those who were challenged produced fewer CRAT solutions than those who were not challenged), this was for true and false memories and did not vary across time.

In another experiment, Brainerd and Reyna (2018) had participants learn, with examples, that the test would include old words, new words that were semantically related to the old words (new similar), and new words that were semantically unrelated to the old words. These examples would essentially serve the same purpose as a warning, because participants were informed that words similar to old words would be on the test, but they were in fact new words. For lists with low association strength, participants were slightly more likely to judge new similar words as new than old but their ability to distinguish between new similar and old dissipated across a 10-day delay.

The limited research on the effect of warning or feedback between testing sessions suggests that general warnings may reduce false memories, but this may be due to a criterion shift. Warnings specifically targeting false memory may be less effective than those targeting how one responds more generally to associated items. The latter will reduce reporting of targets and critical lures to an equal extent. The decreased ability for the warning to help distinguish between old and new across a 10-day delay is consistent with Fuzzy Trace Theory, because it presumably results from decay of the verbatim trace.

### Valence

The emotionality of the lists may influence the trajectory of targets and critical lures in a DRM paradigm. In a study conducted by Howe et al. (2010), participants studied six neutral lists and six negative emotional lists (Experiment 3) and completed a recognition test either immediately or after a 1-week delay. At initial test, participants recognized negative critical lures more often than neutral critical lures. Across delay, target recognition declined more for negative stimuli than for neutral stimuli; however, critical lure recognition remained stable for neutral stimuli and increased for negative stimuli. These results show delayed inflation of critical lures for negative emotional stimuli.

Similarly, in a study by Knott and Shah (2019), participants showed delayed inflation of critical lures for negative stimuli compared to neutral stimuli, when presented quickly. For critical lure recognition that had been presented slowly, participants were more likely to say old to negative words than neutral words. The results from Howe et al. (2010) and Knott and Shah differ from those of Choi et al. (2013) who found that target recognition was higher for negative stimuli than neutral stimuli and found no effect of valence on critical lure recognition. The effect of valence on target recognition was present after a 24-h delay, but critical lure recognition was more frequent for neutral than emotional stimuli (Experiment 2).

Individual differences might moderate the delayed inflation of critical lures for negative stimuli. Norris et al. (2019) found increased memory for negative lists at immediate test for target and critical lure recognition. However, after a 24-h delay, the researchers observed that those low in neuroticism no longer showed increased critical lure recognition for negative stimuli. Valence of the stimuli may also interact with mood, and there could also be a mood congruency effect (Knott and Thorley, 2014; Packard et al., 2014). Knott and Thorley (2014) observed that after a delay, critical lure remember judgments were higher for negative stimuli than neutral stimuli, but only among participants who had watched a video aimed at eliciting a negative mood state.

Conclusions from Choi et al. (2013) and Norris et al. (2019) must be drawn cautiously because delay was compared across experiments. However, it appears that stimulus valence could influence the trajectory of critical lure memory in the DRM paradigm, with some studies showing inflated critical lures for negative words (Howe et al., 2010; Knott and Shah, 2019; Norris et al., 2019). This effect is likely moderated by several factors, including individual differences and mood congruency.

### Developmental Trajectories of the Deese/Roediger–McDermott Paradigm

Many studies have observed a developmental reversal of the DRM effect, where false memories are higher for young adults compared to children (e.g., Brainerd et al., 1995, 2004; Dewhurst and Robinson, 2004; Howe et al., 2004; Howe, 2005; Lampinen et al., 2006; Dewhurst et al., 2007; Anastasi and Rhodes, 2008; Calado et al., 2019). Fuzzy Trace Theory predicts that false memories will increase with age, because children have yet to develop the same extensive level of semantic networks that adults possess (Brainerd et al., 2006).

Unlike adults, children who do not spontaneously generate gist memories should show lower levels of false memory persistence (Brainerd et al., 2006). Additionally, children may not exhibit the delayed inflation effect, because it requires processing gist memories of list themes during recall. Brainerd et al. (2006) examined the effects of immediate and delayed testing across 6- and 11-year-olds. In session one, participants studied the first eight DRM lists, followed by either 2 min of free recall or a distractor task. Participants proceeded with immediate testing, where they received a recognition test consisting of the previously studied lists and eight additional ones. After a 2- to 3-day delay, participants completed a 128-item recognition test, including half the items presented from session one. Older children showed the delayed inflation effect, while younger children did not. While false memory declined over a 2- to 3-day delay, false memory was higher for older children than younger children, regardless of immediate or delayed testing. These findings are consistent with Brainerd et al. (1995), where 5- and 8-year-olds completed recognition tests in an immediate and 1-week delayed testing session. False memories were higher in older children compared to younger children, although some false alarms and hits were persistent across this delay.

Theoretical explanations for these findings on false memory development include Fuzzy Trace Theory (Reyna and Brainerd, 1995; Brainerd and Reyna, 1998). Given that gist-based memories have been shown to be more stable than verbatim memories, true memory should decrease following a delay, while false memory should remain constant (Kintsch et al., 1990; Reyna and Kiernan, 1994, 1995). This process is known as delayed stability and has been demonstrated in children and adults (e.g., Payne et al., 1996; Brainerd et al., 2001, 2006; Howe et al., 2010). Moreover, studies have shown an increase in levels of false memory for critical distractors (i.e., delayed inflation) in adults and children on delayed memory tests (e.g., Brainerd and Reyna, 1996; Payne et al., 1996).

Findings which show delayed inflation of critical lures in older but not younger children are consistent with Fuzzy Trace Theory. Younger children may differ from older children and adults in terms of gist processing. Therefore, it might be expected for critical lures to persist less so in younger children (Brainerd et al., 2006). However, the finding could also be consistent with Associative-Activation Theory. If younger children have less developed associative networks, this would also explain developmental differences with respect to persistence of critical lures across a delay. Additionally, some research shows that when given developmentally appropriate lists, younger children’s memory of critical lures resembles that of older children and adults (Metzger et al., 2008).

### List Connectivity

Norming studies show a wide range in rates of false memories produced by the different DRM lists. Stadler et al. (1999) observed that critical lure recognition varied from 27 to 84% on 36 lists and Roediger et al. (2001b) found that critical lure recognition rates varied from 11 to 84% on 55 lists. Thus, characteristics of the list items, including the number of items in a list, their connectivity to one another, and connectivity to the critical lure, may also influence the trajectory of true and false memories over delays. Connectivity levels refer to the mean connections per associate to the critical lure. Research shows that the connectivity levels of the lists may differentially affect the trajectory of target and critical lure recall across a 1-week delay (Goh and Khoo, 2007). In this study, participants viewed 24 lists, of which half were high connectivity (mean connections > 2), and the others were low connectivity (mean connections < 1). During Test 1 (immediately following study), connectivity facilitated true recall: Memory for targets was higher for high connectivity lists. However, connectivity did not affect critical lure memory. During Test 2 (1 week following), connectivity no longer influenced memory for targets; rather, it influenced memory for critical lures: False memory of critical lures was greater for lists with low connectivity than lists with high connectivity (Goh and Khoo, 2007). This is the only study that we found that used delay and connectivity strength to directly test Fuzzy Trace Theory and an alternative theory (PIER 2). Goh and Khoo (2007) argued that the findings are inconsistent with Fuzzy Trace Theory because high, not low connectivity, should result in greater gist extraction. A stronger gist should lead to inflation of critical lures. The researchers observed the inverse of this, with greater critical lure memory for low connectivity lists, after the delay.

McEvoy et al. (1999) showed that connectivity to the critical lure increased the likelihood that the critical lure will be falsely recalled. However, high connectivity between the presented words within a list, increased target recall. Likewise, critical recall decreased in lists with high connectivity between list words; the effect was consistent across a 1-min and 5-min delay. The explanation for this finding is that access to the true presented words competes with the false recall of the critical lure and serves a protective function. Consistent with this idea, several studies have shown that *recall* rates for the target and the critical lures are inversely related (Stadler et al., 1999; Roediger et al., 2001b). However, other studies have reported positive relationships between targets and critical lures (Brainerd et al., 2003; Cody et al., 2015). Importantly, for recognition, high list connectivity resulted in more true recognition rates (hits) but also more false recognition rates (McEvoy et al., 1999). This points to different underlying mechanisms for recognition and recall of false memory in the DRM paradigm.

## General Discussion

We began this systematic review with one overarching question – when a person develops a false memory for an item in the DRM, how long will that false memory last? Answering that question led us to examine (1) the trajectory of memories for targets and critical lures in the DRM across delay; and (2) the proportion of true versus false memories across delay. The results of our search led to an inconsistent data pattern. However, some findings were consistent across studies. Firstly, false memories for critical lures develop rapidly. The small body of literature that examines at least two testing points within a single session shows that individuals will falsely recognize (Olszewska et al., 2015) within 3–4 s and falsely recall (McDermott, 1996) critical lures within 30 s of the study session. Secondly, target memory begins to decline rapidly. Most studies show steep declines up to about 2 days, entirely consistent with Ebbinghaus’s (1913) forgetting curve. Thirdly, in delays up to 2 weeks, individuals commonly recall proportionately more critical lures than targets. Despite the inconsistency in the trajectory, there is remarkable consensus that across delays critical lures are falsely recalled at higher rates than recalled targets. This occurs with delays ranging from 1–2 days (McDermott, 1996; Thapar and McDermott, 2001; Pardilla-Delgado and Payne, 2017, Experiment 1) to 2 weeks (Toglia et al., 1999) to 2 months (Seamon et al., 2002). Finally, when recall tests are given, target memory generally declines more rapidly than false memory for critical lures.

These similarities in the data are met with an equal number of dissimilarities. There are several factors that help to explain the dissimilarities:

(1) Whether delay was manipulated within or between subjects. For example, Toglia et al. (1999) observed that across three testing periods (immediate, 1 week, or 3 weeks) target recall decreased while critical lure recall remained stable. However, Thapar and McDermott (2001, Experiment 1), who tested participants either immediately, 2 days, or 7 days later, found that both target recall and false recall of critical lures decreased across delay. Failure to observe stability of critical lures in Thapar and McDermott could be due to delay being manipulated between subjects. Furthermore, some researchers compared across experiments to draw conclusions about delay (Choi et al., 2013; Norris et al., 2019). Cross-experiment comparisons are not as methodologically sound as within-experiment comparisons; thus, we urge caution when interpreting results from cross-experiment comparisons.
(2) The number of lists used. This will affect the study and test phase. More items create more interference. This may be particularly relevant when recognition is the dependent variable. For example, Payne et al. (1996) observed stability of critical lure recognition after a 24-h delay, while other researchers did not observe this stability (e.g., Lampinen and Schwartz, 2000). Payne et al. had 16 lists and a 384-item recognition test, while other studies used fewer lists.
(3) The modality of the presentation of items at study and test. Lists presented visually may increase false recognition of critical lures in short-term memory, while lists presented auditorily may increase false recognition of critical lures in long-term memory (Olszewska et al., 2015).
(4) Whether participants recalled items prior to delayed testing sessions. Some studies, even those which used remember/know judgments or recognition tests across two time points, had participants recall the words either directly after each list, or after all lists had been presented. The inclusion of a recall test may influence the trajectory of targets and critical lures. False recall of a specific critical lure on a test increases the likelihood that the word will be falsely recalled on a subsequent test. Increased likelihood to falsely recall critical lures at Test 2 when they were recalled at Test 1 could also be due to forgetting that comes from retrieval. Retrieving information increases subsequent memory for items that were retrieved, while impairing memory for semantically related items that were not retrieved (Anderson et al., 1994). This has been shown to occur with both targets and critical lures (Bäuml and Kuhbandner, 2003).

### Is Persistence of False Memory for Critical Lures Due to a Criterion Shift?

A reviewer sagely asked whether persistence in false memory of critical lures across delay is a result of a criterion shift. That is, with a delay, individuals might be more likely to say they recognize words as having been presented at study. There are few studies which give warning or feedback between testing sessions. The available literature suggests that, compared to specific warnings about critical lures, general warnings (e.g., saying old to any related words) may be more effective at reducing false memories (Miller et al., 2011). General warnings, however, likely decrease reporting of targets and critical lures. This could suggest that individuals are developing a more liberal criterion across delay which might explain instances of delayed inflation of critical lures. If delayed inflation of critical lures results from a criterion shift, it could be argued that individuals would also be more likely to show increased recognition for targets and foils across a delay. Generally, target memory begins to decrease rapidly, even in studies where false memory for critical lures remains stable or increases across time. When examining the effect of delay on foils, there is an important consideration: While most studies included foils, not all studies included analyses of the effect of delay on foils. Comparing patterns of critical lures to foils across delay would help DRM researchers determine whether persistence of critical lures is simply due to a criterion shift.

Studies that examined effect of delays on foils showed that individuals made more errors on related words (critical lures) than unrelated words (foils; Olszewska et al., 2015). Seamon et al. (2002) examined the trajectory of foils and reported a marginal effect (*p* < 0.07); more foils were reported at the 2-month test than the 2-week and immediate test. If this trend is to be interpreted, it could be that participants are adopting a more liberal criterion; however, it should be noted that critical lures decreased from the 2-week to 2-month period, and targets decreased from immediate to the 2-week period. If participants were more liberal in their responding, one might expect similar patterns for all item types. Seamon et al. (2002) attempted to equate baseline recall by analyzing adjusted recall proportions. To do this, they statistically adjusted both targets and critical lures to equal at baseline. They did this to address the concern that a linear scale of recall may not be appropriate for two functions that vary at baseline (e.g., Loftus, 1985; Thapar and McDermott, 2001). These adjusted values resulted in a similar pattern of results (at immediate test, target recall exceeded false recall of critical lures, but false recall of critical lures exceeded target recall at 2 weeks and 2 months). Additionally, adjusted scores showed a decrease in target recall and no change on critical lures from immediate to 2 weeks. From 2 weeks to 2 months there was a slight decrease in target recall and a steep decrease in critical lure recall. Using adjusted scores therefore yields results which are likely not due to a criterion shift.

In sum, because some studies do not analyze or report findings on the effect of delay on foils, it is difficult to rule out that delayed inflation of critical lures is not a result of a criterion shift. Warnings which instruct participants to avoid saying yes to semantically related words, have been shown to reduce both targets and critical lures. This supports a criterion shift argument. However, studies that do report the effect of delay on foils have shown critical lure memory is greater than foil memory. Additionally, it could be argued that a criterion shift would result in delayed inflation of targets as well. This is generally not observed in the literature. The existing research does not appear to support a criterion shift argument, but future research in this area should report the effect of delay on foils.

### Why Are the Results More Consistent With Recall Than Recognition?

Inconsistencies most often seem to arise when dependent variables other than recall (recognition, R/K, lexical decision tasks) are used to examine memory across delay. Why is this so? As with recall, the trajectory of true memories when recognition is used to assess long-term memory is relatively consistent. If a test occurs immediately after study, target recognition is typically higher than false critical lure recognition. Target recognition then declines rapidly, within seconds. Most studies show steep declines in target recognition of up to 2 days. However, the pattern is less consistent when examining the trajectory of long-term critical lure recognition: Some researchers report decreases, some report stability, and some report delayed inflation. As with studies using recall, studies with recognition have highly variable methodologies. Possibly, the task of recognizing targets and critical lures is more sensitive to these variations than is recall. Given that participants view possible targets and critical lures in a recognition task, the number of items at test is a factor that affects recognition but not recall. For example, after a 24-h delay, Payne et al. (1996) observed decreased target recognition and stable critical lure recognition. This study had a recall session preceding the first recognition test, which increased persistence of false memory for critical lures. Payne et al. also had 16 lists and a 384-item test, while other studies which failed to show stability of critical lure recognition had fewer lists and a shorter recognition test (e.g., Lampinen and Schwartz, 2000). Therefore, in addition to factors that influence recall (e.g., within or between subjects, modality of presentation, and whether one completes immediate recall following each list), recognition tasks might be especially sensitive to the number of items during study and test.

### Does Fuzzy Trace Theory or Associative-Activation Theory Better Explain the Persistence of False Memory for Items in the Deese/Roediger–McDermott Paradigm?

Persistence of critical lures across delay has been argued as evidence for Fuzzy Trace Theory. According to Fuzzy Trace Theory, multiple testing sessions promote rehearsal of the gist without rehearsal of the verbatim trace. Increased study sessions allows one to rehearse verbatim traces, thereby countering the stability or inflation of false memories (Brainerd et al., 2003). Studies that examine delay within the same testing session show that false memories for critical lures develop rapidly. This could support gist or semantic encoding even in short-term memory. These findings are somewhat surprising given that at short delays, memory for the original lists should be strong and permit individuals to recognize memory errors of commission. According to Fuzzy Trace Theory, strong verbatim memory allows for rejection of critical lures in the DRM paradigm (Reyna et al., 2016, p. 7). With delays of 3–4 s, the verbatim trace should be available and target recognition should be high. This should decrease the reliance on gist-based processes. In some studies, participants view brief lists and then view a single recognition probe immediately and minutes later. McDermott (1996) and McEvoy et al. (1999; Experiment 3) had participants recall immediately after each list or after a 30–60 s delay. Studies which showed delayed inflation used recognition tests in which participants judged which words on the test appeared in prior lists. It could be that critical lure recognition is more persistent than critical lure recall in short-term than in long-term memory. Overall, the findings from studies examining short-term memory show different trajectories for targets and critical lures. Fuzzy Trace Theory argues that false memory for critical lures is due to reliance on the gist as the verbatim traces decay. After a delay of a few seconds, individuals should have access to the verbatim traces and not have to rely on the gist. Critical lure recall and recognition in such brief periods could support the Associative-Activation Theory of false memory.

The trajectory of true memories when recall is used to assess long-term memory is relatively consistent. Most studies show steep declines up to about 2 days. However, the pattern is less consistent when examining the trajectory of false memory in long-term memory. Some studies show that critical lure recall decreases (McDermott, 1996; Thapar and McDermott, 2001, Experiment 1), others show critical lures remain stable (Toglia et al., 1999), and others show delayed inflation for critical lures (Brainerd et al., 2003; Sherman and Kennerley, 2014). Despite the inconsistency in the trajectory, there is remarkable consensus that across delays critical lures are falsely recalled at proportionately higher rates than recalled targets. This has been shown in delays from 1–2 days (McDermott, 1996; Thapar and McDermott, 2001; Pardilla-Delgado and Payne, 2017, Experiment 1) to 2 weeks (Toglia et al., 1999) to 2 months (Seamon et al., 2002).

The findings of rapid decline of target memory and higher false memory for critical lures compared to target memory after a delay is consistent with the predictions of Fuzzy Trace Theory; that is, the gist is more resistant to decay than is the verbatim trace. The findings could also be consistent with Associative-Activation Theory. This pattern might be especially expected when lists which have high backward association strength are used, which is common in many of the studies that examine delay. Backward association strength has been found to be one of the best predictors of false memory for critical lures (Roediger et al., 2001b). If one presents at study many lists containing targets that are highly associated with the critical lures, this would in theory result in repeated activation of specific critical lures. This pattern would also be expected during a recognition task. Presenting associated words during the retrieval phase would also result in strong activation of the critical lures, activation which might exceed that of the activation for any single target. Therefore, the critical lure could be more activated than specific targets, making the critical lures seem more familiar and more memorable after a delay. Given that the persistence of critical lures could be explained by both theories, further investigation is needed to tease apart whether the effect is due to gist extraction or association activation. Some work has been done to test this. For example, Otgaar et al. (2012) observed that disruptions in the association process through distraction resulted in fewer false memories for critical lures in children.

Findings from McKone (2004) which showed that participants could identify targets more quickly than critical lures on a lexical decision task seem to support Fuzzy Trace Theory which predicts that the verbatim trace and gist representation are similar but distinguishable. Conversely, Sergi et al. (2014) argued for an activation theory of false memory formation in the DRM. This argument is supported by the observation of latency scores on the lexical decision task being equivalent for targets and critical lures, even with a 10-min delay. The differences between McKone (2004) and Sergi et al. (2014) may to be due to how the authors interpreted the data, and the inclusion of an immediate testing condition. Sergi and colleagues observed increased latency between the immediate and 10-min condition; with no immediate condition, McKone could not observe this increase. Additionally, Sergi and colleagues’ argument stems from the similarity of reaction time between targets and critical lures, which were both faster than non-words and new distractors. However, McKone compared reaction time from critical lures associated with presented lists to reaction time from critical lures associated with non-presented lists and found no advantage for critical lures associated with presented lists. So, while it appears that there may be an increase in latency from immediate test to a 10-min delay, targets and critical lures may be distinguished with a lexical decision task. Future research might consider including an immediate delay condition along with a comparison of critical lures from presented and non-presented lists to examine whether the ability to distinguish between targets and critical lures changes across delay.

There is also some evidence that the memory process is different for recognizing targets and recognizing critical lures. Brainerd and Reyna (2018) argued that when participants have the option to respond new-similar to critical lures, the findings indicate that new-similar and old words are remembered in different ways. When judging items as old or new semantically related words, participants were much more likely to correctly judge old words as old, but they judged new semantically related words as old and new semantically related at approximately equal rates. Permitting participants to respond new-similar in addition to old may provide interesting and insightful results if included in future DRM studies. There is also evidence that individuals use memory of targets to help reject critical lures, further suggesting that the two traces may be distinguishable. The idea that individuals will reject a critical lure because they can remember the target word/words is consistent with Fuzzy Trace Theory. If individuals can still access the verbatim then they will be less reliant on the gist.

Lampinen et al. (2005) examined the most common strategies for foils, related associates, and critical lures, even after a 48-h delay. They found that recollection rejection was the most common strategy used for critical lures. This would suggest that individuals still have access to the verbatim trace, for at least some of the targets. This is not necessarily contrary to Fuzzy Trace Theory, because Fuzzy Trace Theory does not stipulate complete degradation of verbatim traces across a specific time. An important finding may be that of different strategies used for related associates and critical lures. Both these items are semantically related to the targets. Therefore, it might be expected that access to the verbatim, recollection rejection, could also be used to reject the unpresented related associates. However, recollection rejection was most common for only critical lures, and distinctiveness was more common for other related associates. One difference between the two non-presented words is the strength of the association between each item and the targets; critical lures have a stronger association. It might therefore be argued that critical lures have a stronger activation than other non-presented associates, a stronger sense of familiarity, and therefore require a certain strategy (e.g., memory for the target) to counter this. Alternatively, other non-presented associates result in a lower level of activation and familiarity, and, thus, can be rejected by strategies other than memory for the targets (e.g., distinctiveness). Therefore, the use of different rejection strategies for critical lures and other non-presented associates is potentially consistent with both Fuzzy Trace Theory and Associative-Activation Theory.

Looking at the subjective experience of remembering across time yields mixed findings. Some studies show that remember judgments for targets and critical lures decrease in a similar way (Lampinen and Schwartz, 2000); other studies show a different pattern for the two (Sherman et al., 2015). Fuzzy Trace Theory suggests that the item-specific information, the verbatim trace, decays rapidly after the study phase. Therefore, we would expect to see that the subjective experience of remembering the target words presented at study would initially differ from the gist-based representation of the critical lure; however, with a delay, the sense of remembering targets and critical lures would be more similar. This data pattern for remember/know judgments occurred in Neuschatz et al. (2001). That said, the subjective experiences for targets and critical lures were differentiated through the memory characteristics questionnaire after a 48-h delay, which is inconsistent with Fuzzy Trace Theory (Neuschatz et al., 2001).

In some studies, false memories are greater for older children than younger children regardless of immediate or delayed testing conditions (e.g., Brainerd et al., 1995, 2006). Additionally, older children will show delayed inflation of critical lures, while younger children do not show this effect. The developmental reversal of the DRM effect can be explained by children’s less extensive levels of semantic networks and a lack of spontaneous, gist memory formation between list targets. Studies which fail to observe critical lure inflation in young children are consistent with Fuzzy Trace Theory. These results are arguably also consistent with the Associative-Activation Theory. Given that younger children have less developed semantic networks, it might also be expected that the presentation of targets results in less activation of critical lures in younger children compared to older children. In fact, dividing attention during the study phase may decrease the spread of activation. This has been shown to decrease false memories for children but not for adults. The explanation for this is that association activation is less automatic for children than adults, making the process more vulnerable to disruption in children (Otgaar et al., 2012, 2019).

Increased memory of critical lures compared to targets across a delay may be consistent with both Fuzzy Trace Theory and Associative-Activation Theory. A sophisticated understanding of how items are associated with one another and with the critical lure might provide more insight into the trajectory of targets and critical lures across various delays. Norming studies show that the rates of memory for critical lures vary across lists. Therefore, the characteristics of the lists themselves influence how they are remembered, the proportion of target and critical lure memories they produce, and how these memories persist across delay. There is good consensus that the strength of the associates to the critical lure increases false memories. However, when examining the association strength between target words rather than between target words and the absent critical lure, lists that have high inter-item association strength produce lower critical lure memory, across delay (McEvoy et al., 1999; Goh and Khoo, 2007). Goh and Khoo (2007) argued that Fuzzy Trace Theory cannot fully explain this finding. Fuzzy Trace Theory predicts that lists with high connectivity, with strong semantic relatedness among the list items, should strengthen the gist representation, thereby increasing recall of the critical lures. Goh and Khoo (2007) argued that the finding of low connectivity having higher rates of critical lure memory is consistent with a specific associative-activation theory of memory (Nelson et al., 1992). Associative-Activation Theory argues that activation of a word increases the likelihood of the word being recalled. Additionally, recall of a list item can cue other list items because of their semantic relatedness. Therefore, high connectivity lists result in greater recall of targets (true memory). However, because high connectivity lists increase the likelihood that recall of one target on the list will cue another target, this decreases the likelihood that the recalled word will cue the critical lure. This is due to the increasing competition of the strong associates that were presented at study. Critical lures would be more likely to be cued through recall where competition from targets is lower, as is the case with low connectivity lists (Goh and Khoo, 2007). Although there were only two studies which examined connectivity strength and delay specifically, the findings are more consistent with Associative-Activation Theory than Fuzzy Trace Theory. Future research on list connectivity across longer delays could provide important insight about the trajectory of targets and critical lures.

In sum, results which show a rapid decrease in target memory and stability or delayed inflation for false memory for critical lures are consistent with Fuzzy Trace Theory. Fuzzy Trace Theory predicts that the verbatim trace, or memory for the presented targets at study, will fade rapidly; conversely, gist, or memory for critical lures, will persist across a delay. This has been shown with studies using recall and recognition. These results, however, may also be consistent with Associative-Activation Theory. This might be particularly true with lists that have a strong backward association strength or high connectivity between the critical lure and the targets. This could result in the critical lure being repeatedly activated to the point where the association is stronger than that of a target that appeared once during study. Studies which show that critical lures are more likely to be judged as remembered rather than known across a delay are consistent with Fuzzy Trace Theory but could also be consistent with Associative-Activation Theory for the same reason described above. Research which shows the rapid generation of critical lure memory may not be consistent with Fuzzy Trace Theory. Reporting of critical lures, or the gist, would not be expected within seconds because the verbatim traces should be available. Finally, research examining the connectivity between items provides insight into the persistence of target and critical lure memory. While strong connectivity between the critical lures and the targets promotes increased false memory for critical lures, strong inter-item connectivity results in decreased memory for critical lures. This has been argued to be inconsistent with Fuzzy Trace Theory; strong inter-item connectivity in theory should promote gist formation, thereby increasing false memory for the critical lure.

### Future Directions

The DRM effect is robust. It is likely because of this that many studies examining interactions do not report the effects on the foils relative to the critical lures. Reporting effects on foils across delay would strengthen the conclusions of the findings and potentially provide useful insight into the mechanisms underlying the DRM. Future research might meta-analyze the effect of delay on targets, critical lures, and foils in the DRM paradigm. Such a study would require authors of original research papers to re-analyze their foil data and then provide their results for the meta-analysis, which is a big ask. Future research could use artificial networks without pre-existing associations, or ones that are seemingly random, to test predictions made by Fuzzy Trace Theory and Associative-Activation Theory. If individuals can be taught, implicitly, to associate non-words to the point where false memories are formed, then this might support Associative-Activation Theory rather than Fuzzy Trace Theory. The key would be to eliminate pre-existing relatedness, and to ensure that individuals were not developing meaning for the associations. Research looking at the effects of distraction on false memory is fascinating, particularly the developmental differences that emerged; distraction was shown to reduce false memory in young children but not adults. Given that spread of activation also occurs at the retrieval phase, future research might consider using a divided-attention task during the retrieval phase of the DRM. Finally, research looking at the connectivity between all items in the paradigm will be useful. If the persistence of false memory after a delay is due to spread of activation, then even subtle activity which serves to activate the critical lure during retrieval (e.g., which item individuals recall first), needs to be examined.

## Conclusion

We conducted our systemic review to answer a broad research question: What is the persistence of false memory for a specific item in the DRM paradigm? To help answer this question we had two research objectives: (1) to examine the trajectory of target memory and false memory for critical lures and (2) to examine whether memory for targets exceeded false memory for critical lures. With respect to (1), the bulk of studies show immediate decreases in target or true memories. The trajectory of critical lures or false memories yields mixed findings, likely due to methodological variation across studies. With respect to (2), the bulk of studies show that across delays of up to 2 months, critical lure memories may be higher than target memories. Despite inconsistent findings, there is enough evidence to conclude that target (true) and critical lure (false) memories behave differently across delay. This pattern is consistent with the Fuzzy Trace Theory prediction that gist processes are more resistant to decay than verbatim memory traces. The pattern is also potentially consistent with Associative-Activation Theory, especially in instances of high association strength. Future research should continue to examine the effect of connectivity on the trajectory of targets and critical lures. A deeper understanding of how remembering specific items serves to cue or inhibit other items in the DRM paradigm will further our understanding of the persistence of false memories in the DRM.

## Data Availability Statement

The original contributions presented in the study are included in the article/Supplementary Material, further inquiries can be directed to the corresponding author.

## Author Contributions

PC developed the conceptualization and research question of the review. KD and IR conducted the article search. All authors summarized articles for the results table. PC and DB completed the initial writing. All authors contributed to the article and approved the submitted version.

## Conflict of Interest

The authors declare that the research was conducted in the absence of any commercial or financial relationships that could be construed as a potential conflict of interest.

## Publisher’s Note

All claims expressed in this article are solely those of the authors and do not necessarily represent those of their affiliated organizations, or those of the publisher, the editors and the reviewers. Any product that may be evaluated in this article, or claim that may be made by its manufacturer, is not guaranteed or endorsed by the publisher.
