# Supplementary material for: The Trajectory of Targets and Critical Lures in the Deese/Roediger–McDermott Paradigm: A Systematic Review
Source: Front Psychol. 2021 Dec 3;12:718818. doi: 10.3389/fpsyg.2021.718818 (PMC8677658; doi:10.3389/fpsyg.2021.718818)
Supplement: Supplementary file 1 [file Image_1.pdf]

Figure 1S

PRISMA Flowchart of Methodology By Identification, Screening and Final Included Studies.

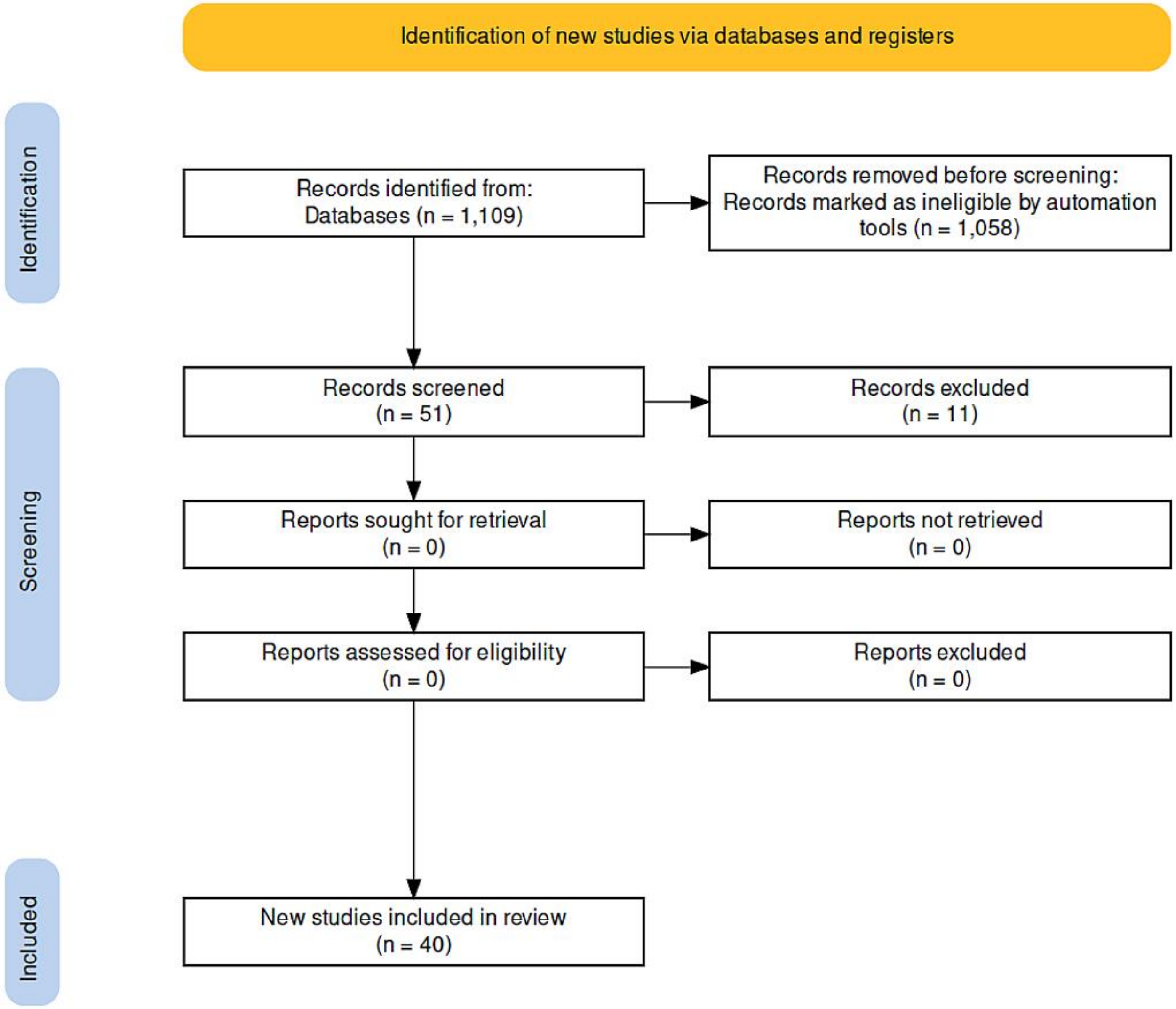

Note. Neal R Haddaway, Chris C Pritchard, Luke A McGuinness. (2021). PRISMA2020: R package and ShinyApp for producing PRISMA 2020 compliant flow diagrams. Zenodo. http://doi.org/10.5281/zenodo.4287834
