# Supplementary material for: The Trajectory of Targets and Critical Lures in the Deese/Roediger–McDermott Paradigm: A Systematic Review
Source: Front Psychol. 2021 Dec 3;12:718818. doi: 10.3389/fpsyg.2021.718818 (PMC8677658; doi:10.3389/fpsyg.2021.718818)
Supplement: Supplementary file 2 [file Table_1.pdf]

Table 1S

Complete list of studies across delay for less than 2-hours, less than 2-days, 2-days, less than 2-weeks and greater than 2-weeks.

< 2 hr Delay	*N*	Design	Method	Recall	Recognition	Remember Know	Other
Brainerd, Payne, Wright & Reyna (2003), Experiment 1	*N* = 131	2(Condition: blocked, random) x 2(Item Type: target, critical lure) x 3(Delay: 1 minute, 3 minutes, 5 minutes) within-subjects design.	*Study phase:*<br>Participants heard 6, 15-word lists.<br>Presentation rate = 2 seconds.<br>*Test 1:*<br>Participants had a 2-minute filled delay, then did 5 minutes of free recall.<br>*Test 2:*<br>Participants did another 2 minutes of filled delay, then 5 minutes of free recall.<br>*Test 3:*<br>Participants did another 2 minutes of filled delay, then 5 minutes of free recall.	*Test 1 (2 minutes)*<br>Targets < Critical lures<br>*Test 2 (9 minutes)*<br>Targets < Critical lures<br>*Test 3 (16 minutes)*<br>Targets < Critical lures<br>• Critical lure recall increased in the 3 memory tests, while target recall remained stable.			
Choi, Kensinger, & Rajaram (2013)<br><br>*Note.* Compares Exp. 1 and 2.	*N* = 48 (Exp. 1)<br>*N* = 48 (Exp. 2)	3(Valence: negative, neutral, positive) x 2(Memory Task: recognition, cued-recall) mixed design, with Memory Task as a between-subjects factor.	*Study phase:*<br>Participants viewed 225 words with corresponding pictures and category names and rated each word based on its goodness of fit.<br>Presentation rate = 6 seconds/ word.<br>*Test 1 (Exp 1):*<br>Following a 30-minute delay, participants either completed an old/new recognition or a cued-recall retrieval task with an additional 135 non-studied items included. For the recognition task, 360 studied and non-studied words were presented without their corresponding pictures and names. For the cued-recall task, category words were listed consecutively so participants could recall as many words as possible.		*Test 1(Exp. 1)*<br>• Targets: negative items > neutral items.<br>• Targets: positive items = neutral items.<br>• Valence had no effect on critical lures.<br>*Test 2 (Exp. 2)*<br>• Targets: negative items > neutral or positive items.<br>• Critical lures: neutral items > negative and positive items.<br>• Critical lures: negative items = positive items.<br>• Critical lures: Main effect of Valence was present in Exp. 2 (1-day delay), but not in Exp. 1 after the 30-min delay.		For both experiments, intrusion rates in cued recall were at floor, so results for this experiment include results for targets and critical lures from the recognition task.

			Participants were then re-presented with the 360 items and rated each item for valence.<br>*Test 2 (Exp 2):*<br>1-day delay.				
Festini & Reuter-Lorenz (2013), Experiment 2	*N* = 56	Single Factor (Delay: 3 seconds, 8 minutes) within-subjects design.	*Study phase:*<br>112, 3-item semantically related lists were used. There were 5 probe types: remember-studied probes (positive), negative probes, remember-related probes, forget-related probes, new-unrelated probes, forget-studied probes. The probe rate was 2/3 negative probes and 1/3 positive probes. 48 trials were presented: 8 for each negative probe type and 16 for the positive probe type. For each trial, participants studied a set of 2, 3-word lists, with 1 list presented on either side of the screen<br>Presentation rate = 3 seconds/ list set.<br>A forgetting cue then appeared for 2 seconds, prompting participants which list they should forget.<br>*Test 1*<br>Following a 3-second delay, participants indicated whether a single recognition probe word was included in the set of to-be-remembered words.<br>*Test 2:*<br>For the Long-Term Memory recognition test, there were 48 trials. Of these trials, there were 8 per probe type, except for 16 forget-related probes. Similar to T1, there		• To-be-remembered lists > to-be-forgotten lists.<br><br>*False alarms*<br>• Unrelated probes < remember—related probes and forget-related probes.<br>• Forget-related probes = new-unrelated probes.<br>• Forget-related probes < remember-related probes.<br>• Forget-studied probes > new-unrelated probes.<br>• Forget-related words > new-unrelated words.<br><br>• Reduced false alarms for items of to-be-forgotten lists in T1 and T2.<br><br>• Remember-related probes (critical lures) T1 < T2.<br>• To-be remembered probes (targets) T1 > T2.<br>*(Taken from Table 1 in Festini & Reuter, 2013)*		• Positive probes: words identified as to-be-remembered words.<br>• Negative probes: words excluded from the set or received a forgetting cue.<br>• Remember-related probe: words semantically related to the to-be-remembered words.<br>• Forget-related probe: words semantically related to the to-be-forgotten words.<br>• New-unrelated probe: words unrelated to the presented words.<br>• Forget-studied probe: words

			were 5 probe types. Participants saw words consecutively for 4 seconds and specified whether they had studied the word, regardless of whether it previously appeared in a to-be-remembered or -forgotten list.				included in the to-be-forgotten words.

Flegal, Atkins & Reuter-Lorenz (2010), Experiment 1	*N* = 27	3(Probe Type: related lure, unrelated lure, target) x 2(Delay: 3-4 seconds, 20 minutes), within-subjects design.	*Study phase*:<br>Participants viewed 128, 4-word lists. 12 trials were associated with a lure (theme words associated with a list), 12 were associated with an unrelated negative probe (theme words from a non-presented list) and 12 were associated with a positive probe (theme words that replaced an item from that list). There was a fourth group of lists that served as unrelated negative probes, but all four groups were counterbalanced. Presentation rate = 1.2 seconds/ 4-item list. After each 4-word list appeared, there was a 3 to 4-second delay, where participants completed a distractor task.<br>*Test 1*: *STM*<br>Following a 3 to 4-second delay, participants indicated whether the probe words that appeared on the screen for 3 seconds were previously in the memory set (i.e., yes/no). Next, participants rated their confidence for their responses. Of the 96 trials, 48 trials were probed at STM, 16 were of each probe		*Test 1*<br>False memory effect for unstudied related-lure probes and unstudied unrelated-lure probes.<br>*Test 2*<br>• False memory effect for unstudied related-lure probes and unstudied unrelated-lure probes.<br>• The rate of false recognition did not increase from STM to LTM.		

			type: related lure, unrelated lure, target.<br>*Test 2: LTM*<br>2 minutes after the STM trials ended (approximately 20 minutes), participants completed a 96-item LTM recognition test that was similar to the one in Test 1.				
Flegal, Atkins & Reuter-Lorenz (2010), Experiment 2	*N* = 32	3(Probe Type: related lure, unrelated lure, target) x 2(Delay: 3-4 seconds, 20 minutes), within-subjects design.	*Study phase:*<br>The same word lists were used from Exp. 1.<br>*Test 1*<br>Remember/know/guess judgments replaced confidence ratings.<br>Presentation rate = 1.2 seconds/ list followed by 3- to 4-second distractor task.<br>*Test 2:*<br>Same as Exp. 1.			• Higher rates of “remembering” target probes than related- or unrelated-lure probes.<br>*Test 1*<br>• Critical lures: studied related-lure probes > unstudied unrelated-lure probes.<br>*Test 2*<br>• Critical lures: unstudied related-lure probes > unstudied unrelated-lure probes.<br>• Remember responses: STM > LTM.<br>• Remember responses: related > unrelated lures at STM and LTM.<br>• No increase in false recognition from STM to LTM.	
McEvoy, Nelson, & Takako Komatsu (1999), Experiment 3	*N = 40*	2(Connectivity of Critical Lures: high,	*Study phase:*<br>Participants heard 12, 15-word lists (6 high	• High connectivity = higher Target recall.			

		low) x 2(Delay: immediate, 1 minute) mixed design, with Delay as the between-subjects factor.	connectivity lists, 6 low connectivity lists). Presentation rate = 2 seconds/ word.<br>*Test 1:*<br>Participants either immediately completed 1.5 minutes of free recall followed by a 1-minute delay or had a 1-minute filled delay followed by 1.5 minutes of free recall.<br>*Test 2:*<br>Participants then had a 5-minute delay and recalled words from all lists.	• Low connectivity = higher False recall of critical lures.<br>*Test 1*<br>• False recall immediate = false recall 1-minute delay.<br>• Target recall immediate > Target recall 1-minute delay.<br>• Connectivity: immediate > 1-minute delay.<br>• High connectivity list words more likely to be recalled.<br>*Test 2*<br>• Critical lures: T1 > T2.<br>• Targets: T1 > T2.<br>• Low connectivity: Critical lures more likely to be falsely recalled in T1 and T2.			
McKone (2004)	*N* = 73	2(Type of Target: lure item, list item) x 2(List Status: studied, unstudied) x 2(Delay: 3 minutes, 10 minutes) mixed design, with Delay as the between-subjects factor	*Study Phase:*<br>Participants viewed 16, 15-word lists. Presentation rate = 1.5 seconds/ word.<br>*Test 1:*<br>Participants either had 3 or 10 minutes to complete a lexical decision task (LDT). The LDT had 64 trials, with 32 words and 32 non-words. The 32 words included 8 targets and 8 critical lures from studied lists, and 8 targets and 8 critical lures from unstudied lists.<br>*Test 2:*<br>Participants completed a 5-minute old/new recognition test containing 32 target words (8 targets and 8 critical lures from each studied and unstudied list).	• No effects of Delay on LDT or recognition.		LTD<br>Reaction Time<br>*Targets*<br>• Studied lists < Unstudied lists.<br>*Critical Lures*<br>• Studied lists = Unstudied lists.	
Miller, Guerin, & Wolford (2011)	*N* = 96	2(Presentation: presented related item, non-presented related item) x 3(Item Type:	*Study phase:*<br>Participants heard 14, 15-word lists. Participants were told to remember the words. Presentation rate = 1.5 seconds/ word. There was a		*Test 2*<br>• Criterion warnings reduced ‘old’ responses and reduced critical lures.		

		critical items, related items, unrelated items) x 3(Warning Group: no warning, strong critical lure warning, criterion warning) mixed design, with Warning Group as the between-subjects factor.	10-second pause between lists.<br>*Test 1:*<br>Following a 2-minute filler, participants completed a 24-item yes/no recognition test, consisting of 6 critical words, 6 related words, and 12 unrelated words. Half of each were presented or non-presented words. Then, participants either received no warning, a strong critical lure warning, or a criterion warning.<br>*Test 2:*<br>Participants completed the same recognition test as in T1.		• Critical lure warnings were slightly better than no warnings at all.<br>• Critical lure warnings did not reduce false alarm rates to critical lures.<br>• Criterion warnings reduced false alarm rates to critical lures.<br>• Criterion warnings reduced false recognition.		
Olszewska, Reuter-Lorenz, Munier & Bendler (2015), Experiment 3	N = 32	3(Probe Type: related negative lures, unrelated negative lures, studied positive probes) x 2(Modality: auditory, visual) within-subjects design.	*Study phase:*<br>96, 4-word lists were presented.<br>*Test 1 (STM):*<br>There were 72 trials (4 blocks of 18 trials, 2 for each Modality). These trials had 3 probe types: related negative lures (non-studied theme words from studied lists), unrelated negative lures (non-studied theme words from non-presented lists) and studied positive probes (theme words presented in studied lists). Participants heard or viewed 4 words appearing consecutively for 1 second each. After the last word, participants recited “the, the, the” for 3 seconds. The probe word then appeared; participants responded old or new.<br>*Test 2 (LTM)*<br>Followed Test 1 which took approximately 20 minutes.		• More errors occurred in delay testing condition than immediate testing condition.<br>• More errors for critical lures than unrelated distractors.<br>*Test 1(STM)*<br>• False recognition: visual modality > auditory modality.<br>• Fewer critical lures when lists were studied and tested in the auditory modality.<br>*Test 2 (LTM)*<br>• Targets: T1 > T2.<br>• False recognition: auditory modality > visual modality.<br>• Fewer critical lures when lists were studied and tested in the visual modality.		

			On half the trials, no probe appeared. These trials were tested in the LTM recognition test. Participants indicated whether the word appeared during the study phase. There were 72 LTM trials, (36 were not tested at STM). LTM trials were divided into two modality blocks (Auditory and Visual). Probes appeared in the same modality in which they were studied.				
Sergi, Senese, Pisani & Nigro (2014)	*N* = 100	5(Word Type: word lists, non-word lists, critical lures, matched words, matched non-words) x 4(Delay: immediate, 3 minutes, 10 minutes, control) mixed design, with Delay as the between-subjects factor.	*Study phase:*<br>Participants viewed 135 items (5, 15-word DRM lists and 4, 15-non-word lists, Presentation rate = 2 seconds/ word.<br>*Test 1:*<br>Participants completed a lexical decision task (LDT) immediately (no filler)<br>*Test 2:*<br>Participants completed the LDT after a 3-minute filled delay.<br>*Test 3:*<br>Participants completed the LDT after a 10-minute filled delay.<br>Test 4:<br>Participants completed the LDT directly after the filler (control). Time of filler was not reported.		LDT latency:<br>• Target latency = critical lure latency.<br>• Targets and critical lures < non-words and matched new words.<br>• In the control condition, there were no latency differences between targets, critical lures; new words were shorter than non-words.<br>*Test 3:*<br>• Target latency T3 > T1.<br>• Critical lure latency T3 > T1.		
**< 2-day Delay**	***N***	**Design**	**Method**	**Recall**	**Recognition**	**Remember Know**	**Other**
Brainerd, Forrest, Karibian & Reyna (2006), Experiment 1	*N* = 120	2(Age: 6-year-olds, 11-year-olds) x 2(Order of Recall Testing: recall first, no recall first) x	*Study phase:*<br>Participants heard 16, 12-word lists. Following the presentation of 8 lists, participants either completed 2 minutes of oral free recall or a distractor task.		*Test 1*<br>• 6-year-olds did not show list-strength effect, but 11-year-olds did.<br>• 6- and 11-year-olds falsely recognized		

		2(Strength: strong lists, weak lists) x 2(Delay: immediate, 2-3 days) x 2(Type of Memory Response: true, false) factorial design, with Age and Order of Recall Testing as the between-subjects factors.	*Test 1:*<br>Participants completed an auditory 64-word old/new recognition test from the recently presented lists.<br>*Test 2:*<br>A 128-word recognition test was administered after a 2- to 3-day delay. This test included the 64 words from T1. T1 participants were tested on 32 targets, 8 critical distractors and 8 semantically related distractors. These items were not evaluated on T1. 16 distractors were unrelated to any of the tested lists.		critical distractors for lists which had been recalled prior, but effect was larger for 6-year-olds.<br>• Critical lures: strong list critical distractors > weak list critical distractors in 11-year-olds.<br>• Prior recall of lists had no effect on performance in 11-year-olds.<br>• Targets and Critical Lures: Prior recall of lists had no effect on targets or related distractor critical lures in 6-year-olds.<br>• Prior recall increased critical lure rate for critical distractors in 6-year-olds.<br>*Test 2:*<br>• Targets: T1 > T2.<br>• Critical lures: T1 > T2.<br>• 11-year-olds: targets for prior recall tests > no prior recall.<br>• 6-year-olds: list strength had no effect on targets or critical lures for semantically related distractors.<br>• Critical lures for critical distractors: strong lists > weak lists in 11-year-olds.<br>• Prior recognition testing increased targets and critical lures of critical distractors in 6- and 11-year-olds.<br>• Prior recognition testing increased		

					critical lures of related distractors in 11-year-olds.<br>• Prior recognition tests:11-year-olds > 6-year-olds.<br>• False memory increased between 6- and 11-year-olds.		
Brainerd, Reyna, & Brandse (1995), Experiment 1	*N* = 61	Single factor (Age: 5-year-old, 8-year-old) between-subjects design.	*Study phase:*<br>Children heard 60 familiar nouns.<br>Presentation rate = 3 seconds/ word.<br>*Test 1:*<br>This was followed by a 5-minute filler and then a yes/no recognition test. The recognition test included 30 targets and 30 distractors.<br>*Test 2:*<br>1 week later, children completed the same recognition test.		• Critical lures were higher than targets for older children compared to younger children.<br>• Persistence rates for targets > persistence rates for critical lures in 5-year-olds; opposite for 8-year-olds.<br>*Test 2*<br>• Initial critical lures persisted over 1-week delay and could be as persistent as targets; 8-year-olds > 5-year-olds.		
Brainerd, Reyna, & Brandse (1995), Experiment 2	*N* = 120	2(Age: 5-year-old, 8-year-old) x 2(Item Type: categories, rhyme) x 2(Delay: immediate, 1 week) mixed design, with Age and Item Type as the between-subjects factor.	*Study phase:*<br>The procedure resembled Exp. 1, except children studied 64 words. 32 of the originally presented words were replaced by 16 unrelated distractors and 16 words from the categories (e.g., red- colour) or rhymes (e.g., red-bed).<br>*Test 1*<br>Half the participants from each age level completed the categories condition and half completed the rhymes condition. Participants completed a 64-item yes/no recognition test.<br>*Test 2*		*Test 2*<br>• Initial critical lures and targets persisted for younger and older children in both the categories and rhyme conditions.<br>• Critical lures to target-related distractors > targets.<br>• Higher critical lures in the categories and rhyme conditions compared to targets for younger and older children.<br>• No difference in critical lures to nominally unrelated distractors and targets in either		

			1-week later, participants completed another recognition test.		condition for all children.<br>• Persistence rates for rhymes and nominally unrelated distractors were higher in older children compared to younger children.<br>• Persistence rates for categories did not increase with age, but values were near ceiling. In Exp. 1, age increases in target rate persistence was reliable (both conditions).<br>• Targets persistence (categories and rhyme conditions): younger children > older children.<br>• Persistence rates for nominally unrelated distractors and the rhymes increased with age.		
Brainerd, Reyna, & Brandse (1995), Experiment 3	*N* = 60	2(Age: 5-year-olds, 8-year-olds) x 2(Item Type: familiar nouns, nonsense words) x 2(Delay: immediate, 1 week) mixed design, with Age as the between-subjects factor.	*Study phase:*<br>5- and 8-year-olds heard 64 words. Half these words were familiar nouns that were previously used; half were 1- to 2- syllable nonsense words.<br>*Test 1:*<br>Participants completed an immediate recognition test, where half the familiar nouns were replaced by distractors, and half the nonsense words were replaced by nonsense distractors. Half the nonsense distractors rhymed with the replaced target nonsense words.<br>*Test 2:*		*Test 1:*<br>• Initial critical lures were higher for related distractors compared to nonsense distractors for all age groups.<br>*Test 2:*<br>• Critical lures to rhyming nonsense distractors = critical lures to unrelated nonsense distractors.<br>• Targets to nonsense targets > critical lures to nonsense distractors.		

			1week later, participants completed another recognition test.				
Goh & Khoo (2007)	*N* = 94	2(Connectivity: high, low) x 2(Recall Type: veridical, false) x 2(Delay: immediate, 1 week) mixed design with Delay as the between-subjects factor.	*Study phase:*<br>Participants viewed 24 lists consisting of one critical lure and 10 associates [24, 10-word lists].<br>Presentation rate = 1 second/ word.<br>*Test 1:*<br>Participants immediately wrote down as many words as they could remember for 10 minutes.<br>*Test 2:*<br>1-week later, participants wrote down as many words as they could remember for 10 minutes.	*Test 1*<br>• High connectivity lists increased target recall compared to low connectivity lists; connectivity had no effect on critical lure recall.<br>*Test 2*<br>• Low connectivity lists facilitated critical lure recall compared to high connectivity lists. Connectivity was not reliable for target recall.			
Houben, Otgaar, Roelofs, Smeets & Merckelbach (2020)<br><br>*Note.* Delay was manipulated across experiments.	*N* = 72 (Exp. 1)<br>*N* = 68 (Exp. 2)	2(Condition: eye movement, control) x 2(Valence: neutral, negative) x 2(Delay: immediate, 2 days), mixed design with Condition and Delay as the between-subjects factors.	*Study phase:*<br>Participants completed the Dissociative Experiences Scale (DES) and viewed 5 neutral and 5 negative DRM lists. Each list included 10 associates.<br>Presentation rate = 2 seconds/ word. Each word appeared for 2 seconds. Participants then completed a 5-minute filler, and then a free recall task. During free recall, participants completed an eye movement task (eye movement condition) or watched a blank screen (control).<br>*Test 1 (Exp 1):*<br>Following free recall there was a 5-minute filler and then a 78-item recognition test. The recognition test contained 40 correct items, 10 critical lures, 10 non-presented related items, and	*Test 1 (Exp. 1)*<br>• Neutral lures > negative lures<br>*Test 2*<br>• Neutral words: eye movement > control; no difference for negative words.<br>• Critical lures: eye movement > control<br>• Targets (eye movement and control): Immediate > 2 days.<br>• Critical lures: (control) Immediate > 2 days.<br>• Intrusions immediately < 2 days.	*Test 1*<br>• Targets and critical lures: negative know responses > neutral know responses.<br>*Test 2*<br>• Targets (eye movement and control group): Immediate > 2 days.<br>• Critical lures: Immediate = 2 days.<br>• Critical lures: negative words > neutral words.<br>• Critical lures: eye movement > control.<br>• Recognized negative > neutral critical lures.<br>• Related words: eye movement > control.<br>• Unrelated words: eye movement > control.		

			18 non-presented unrelated items. Participants also provided remember/know responses.<br>*Test 2 (Exp 2)*:<br>Participants returned 2 days later to complete the same free recall and recognition test.				
Huff, Coane, Hutchison, Grasser & Blais (2012), Experiment 2	*N* = 80	2(Initial Task: recognition, math) x 2(List Type: direct lure, mediated lure) mixed design, with Initial Task as the between-subjects factor.	*Study phase:*<br>Participants read 6, 15-word lists and completed a 6-word recognition test or a filler after each list.<br>Presentation rate = 1.5 seconds/ word.<br>*Test 1:*<br>After all 6 lists, half the participants completed a 36-item old/new recognition test. Participants repeated the above on another 6 lists. The remaining participants completed a 60-second filler.<br>*Test 2*<br>Participants completed an old/new recognition test.		*Test 1 (Uncorrected)*<br>• Targets > Critical lures.<br>*Test 2 (Uncorrected)*<br>• Targets = Critical lures.<br>• Targets: T1 > T2.<br>• Critical lures : T1 = T2.<br>*Test 1 (Corrected)*<br>• Targets > critical lures.<br>*Test 2 (Corrected)*<br>• Targets = critical lures.<br>• Targets: T1 > T2.<br>• Critical lures : T1 > T2.		• Note: All results here reported for Direct items, which are standard DRM lists [results largely same when combining Direct and Mediated scores].<br>• Corrected Accuracy: targets to studied items minus critical lures to non-studied control items Corrected critical lures: FA rates to studied critical lures minus FA rates to non-studied critical lures).
McDermott (1996), Experiment 1	*N* = 45	3(Delay: immediate, 2 days, no test) x 3(Recall:	*Study phase:*<br>Participants heard 24, 15-word lists.	*Test 1*<br>• The 30-second delay had no effect on recall of the			

		immediate, 30-second, none), within-subjects design.	Presentation rate = 1.5 seconds/ word.<br>*Test 1:*<br>After each list was presented, participants either (1) took an immediate recall test (90-seconds recall followed by 30 seconds of math problems), (2) took a delayed test (30 seconds of math problems followed by 90-second recall), or (3) took no test (30 seconds of math problems followed by an additional 90 seconds of math problems).<br>*Test 2:*<br>After the last list was presented, participants were told to return after 2 days. Participants were given 15 minutes to recall as many words as they can from the study phase.	critical items as compared to the immediate condition.<br>• For studied items, the effect of recall in the 30-second delay condition and the immediate condition were reliable, with more words recalled in the immediate condition.<br>• Accurate recall > false recall. After the 30-second delay, no difference between accurate and false recall.<br>*Test 2*<br>• Targets and critical items recalled after 2 days were lower.<br>• Critical items recalled exceeded studied items recalled.<br>• Targets: Participants in the immediate testing condition in T1 recalled more targets than participants in the delayed testing condition in T1.<br>• Critical lures: immediate = 2 days.<br>• Critical lure recall proportions exceeded target recall proportions in all conditions for almost all serial positions.<br>• Critical non-presented items > targets.			
McDermott (1996), Experiment 2	*N* = 40	2(Ordering: blocked, random) x 6 (Test Session: Trials 1 – 5, Day 2) x 2 (Item Type: studied, critical) mixed design with Ordering	*Study phase:*<br>6, 15-word lists were divided into two 45-word lists of three 15-word associative sets per list.<br>Presentation rate = 1 second/ word.<br>*Test 1*<br>Participants heard one list five times, and each	*Test 1*<br>• Recall increased for targets across trials.<br>• Critical lure recall: blocked > random.<br>• Critical lure recall decreased across trials but was not eliminated. In Trial 1, critical items recalled exceeded the studied items,			

		as the between-subjects factor.	presentation was followed by a 4-minute recall test.<br>*Test 2*<br>Participants completed a 12-minute recall test 1 day later.	but this pattern was reversed by Trial 3.<br>*Test 2*<br>• Recall for studied items decreased.<br>• Recall for critical lures increased.			
Neuschatz, Payne, Lampinen, & Toglia (2001), Experiment 1	N = 138	2(Item Type: studied, critical non-presented) x 3(MCQ Rating: all items, remembered items, MCQ only) x 2 (Delay: immediate, 2 days) mixed design, with MCQ Rating and Delay as between-subject factors.	*Study phase:*<br>Study list consisted of 10 auditory sub-lists of 10 items selected from 24 lists. Presentation rate = 2 seconds/ word.<br>*Test 1*:<br>Participants completed a 5-minute filler task.<br>Immediate: participants completed an old/new recognition test. 80 words appeared in same order. 4 types of items appeared: studied items, critical non-presented items, distractors, and critical non-presented distractors. ‘All Items’ and ‘Remembered Items’: participants provided remember/know judgements for words they said were ‘old.’<br>Memory Characteristics Questionnaire (MCQ) Only: participants completed a 2-minute filler.<br>All Items and MCQ Only: Participants completed the modified version of the MCQ for every item they labelled as “old.” Remember Items: participants completed the modified version of the MCQ for items they had labelled a “remember”; later they completed the MCQ for the “know” items. All		*Test 1:*<br>• Accuracy was higher in the immediate testing condition.<br>• Accuracy: targets > critical non-presented items.<br>*Test 2:*<br>• ‘Old’ items: T1 > T2.<br>• Participants were more likely to call critical lures “old” than distractors or critical non-presented distractors for the immediate and delayed conditions.<br>• Studied items received higher MCQ ratings than critical non-presented items for auditory detail and feelings for both T1 and T2.<br>• After 2 days, participants remained able to discriminate targets and critical lures.	• Remember responses: critical lures > distractors and critical non-presented distractors.<br>*Test 2*<br>• Participants judged more targets than critical non-presented items as ‘remembered’ items in the immediate testing condition but not in the delay testing condition.<br>• Participants made just as many “remember” responses for targets as they did for critical lures.	

			participants then completed a questionnaire about the filler article.<br>*Test 2*:<br>2 days after filler task Participants in the Delayed Test condition completed the recognition test, the remember/know task, and the modified version of the MCQ according to their assigned condition. Procedures were identical to T1.				
Neuschatz, Payne, Lampinen, & Toglia (2001), Experiment 3	*N* = 131	2(Item Type: critical, non-presented) x 3(Warning Instructions: none, moderate, strong) x 2(Delay: immediate, 2 days) mixed design, with Warning and Delay as the between-subject factors.	*Study phase:*<br>Procedure was the same as Exp. 1, but warnings were given before the recognition test and the source attribution task.<br>*Test 1:*<br>A 5-minute filler task followed. Participants in the immediate testing condition were told the warning appropriate to their condition. Participants then completed the old/new recognition test used from Exp. 1, followed by a source attribution task.<br>*Test 2*:<br>2 days after the filler task, participants were told the appropriate Warning instructions that were followed by the recognition test and source attribution task.		• Warnings failed to reduce overall rate of true and false memories.<br>• Warnings did not affect participants' source attributions for studied items.<br>• Strong warning reduced source attributions for critical non-presented distractor items.<br>*Test 1*<br>• Participants attributed fewer items to a source in the strong warning condition than they did in the no warning and moderate warning conditions.<br>*Test 2*<br>• Targets: immediate > 2 days.<br>• Critical lures: 2 days > immediate.<br>• Accuracy: immediate > 2 days.<br>• Source attributions: immediate > 2 days.		Source attribution task: Participants decided whether male or female read the words that they indicated were 'old' and if they did not know, they guessed '?'.
Norris, Leaf, & Fenn (2019)	*N* = 138	Single Factor 2(Valence: negative,	*Study phase:*<br>Visual lists		*Test 1 (Ex. 1)*<br>• Targets: negative > positive.		

Note. Delay was manipulated across experiments.	(Exp. 1)<br>*N* = 100 (Exp. 2)	positive) within-subjects design.	10 negative and 10 positive lists (10 words each). Presentation rate = 1.5 seconds/ word.<br>*Test 1 (Exp. 1)*<br>Following a 3-minute filled delay, participants completed a 160-item old/new recognition test (60 targets and 20 critical lures from studied lists). Participants then completed The Big 5 Factors of Personality scale.<br>*Test 2 (Exp. 2)*<br>Design and procedure of Exp. 2 was identical to Exp. 1, except Exp. 2 had a 1-day delay.		• No effect of neuroticism.<br>• Critical lures: negative > positive.<br>• Participants responded faster (inaccurately) to negative critical lures than positive critical lures.<br>• Higher false recognition for critical lures than for list words from unstudied lists.<br>• Higher false recognition for negative unstudied words than for positive unstudied words.<br>*Test 2 (Ex. 2)*<br>• Targets: negative words > positive words.<br>• Low neuroticism: negative critical lures = positive critical lures.<br>• High neuroticism: negative critical lures > positive critical lures.<br>• Participants were faster to respond that they had seen negative critical lures than positive.<br>• Critical lures > list words from unstudied lists.<br>• False recognition: negative unstudied words > positive unstudied words.<br>• Participants responded faster (inaccurately) to negative than positive unstudied list words.<br>• Neuroticism moderated the effects of false memory for negative and positive		

					information after a 1-day delay.		
Pardilla-Delgado & Payne (2017)	*N* = 117	2(Order: SLEEP 1st, WAKE 1st) x 2 (Delay: 1 day, 2 days) x 3(Memory type: true, false, intrusions), with Delay as the between-subjects factor.	*Study phase:*<br>Participants heard 16, 12-item lists.<br>Presentation rate = 2 seconds/ word. Participants completed the study phase at 9:30am.<br>*Test 1*<br>Half the participants returned 1 day later to complete a free recall task, immediately followed by an old/new recognition task. The recognition test included 96 words (48 targets, 16 critical lures from studied lists, and 32 unrelated foils from unstudied lists).<br>*Test 2*<br>The remaining participants returned 2 days later to complete the same tasks from Test 1.	• No main effects or interactions of delay on recall.<br>• Targets and critical lures: SLEEP 1st > WAKE 1st.<br>• Planned comparisons were conducted with low and high performers. This was determined by a median split on target scores.<br>• Participants who slept first had an increase in false recall, but only true for low performers not higher performers.	• No main effects or interactions involving delay on A’ emerged.<br>• Critical lures > Targets.<br>• Participants who slept first recognized more targets than participants who were awake first.<br>• Critical lures: SLEEP 1st = WAKE 1st.<br>• Participants were more liberal in their responding to critical lures than targets.<br>• SLEEP 1st participants were less biased in their responding than WAKE 1st participants. This effect occurred for targets and critical lures.<br>• SLEEP 1st participants had increased memory discriminability (A’) for studied (true) words, and decreased bias (B”) for both studied and non-studied, critical (false) words.		• *A’* was used to measure recognition.<br>• *B”* was used to measure response bias.
Parker, Dagnall, & Abelson (2019), Experiment 1	*N* = 150	3(Encoding Condition: survival, moving, pleasantness) x 2(Delay: 5 minutes, 1 day) between-subjects design.	*Study phase:*<br>Participants read 14, 10-word lists divided into 2 groups of 7 lists (list A and B), so half the participants read list A and half read list B.<br>Presentation rate = 5 seconds/ word.<br>*Test 1:*<br>Following a 5-minute filler, an old/new recognition test was given with remember-know instructions. The test			*Test 2*<br>• Targets (remember responses): 1 day < 5 minutes.<br>• True recollection: 1 day < 5 minutes.<br>• Overall unrelated critical lure rate (remember responses): 1 day > 5 minutes.	

			was comprised of studied and unstudied lists, each consisting of 7 critical lures and 14 list words from serial positions 4 and 7. If participants responded yes on the recognition test, they were asked to provide a remember//know/guess response.<br>*Test 2:*<br>1-day later, similar to T1.			• Related false memory was enhanced by survival processing and did not decrease over 1 day.<br>• Overall target rate and "remember" responses: 1 day < 5 minutes.<br>• Overall unrelated critical lure and "remember" responses: 1 day > 5 minutes.	
Parker, Dagnall, & Abelson (2019), Experiment 2	*N* = 150	3(Encoding Condition: survival, moving, pleasantness) x 2(Delay: 5 minutes, 1 day) between-subjects design.	The procedure resembled Exp. 1, except: 14, 11-word lists were used. The first word in each list was removed and used as the critical lure, while the remaining words were used as list words.			*Test 2*<br>• Targets (remember/know responses): 5minutes > 1 day.<br>• True recollection and true familiarity: 5minutes > 1 day.<br>• Delay was significant for the moving condition.	
Payne, Elie, Blackwell, & Neuschatz (1996), Experiment 1	*N* = 60	2(Activity: recall, arithmetic) x 2 (Delay: 2 minutes, 1 day) mixed design, with Delay as the between-subjects factor.	*Study phase:*<br>Participants heard 16, 15-word lists.<br>Presentation rate = 1 seconds/ word.<br>After each list, participants recalled the words or completed 2 minutes of arithmetic problems.<br>*Test 1:*<br>Participants completed a 384-item old/new recognition test (240 items		• Targets and critical lures decreased across delay. The decrease was greater for targets than critical lures.<br>• Recognition rates were lower for lists followed by arithmetic than lists followed by recall. This effect was greater for targets than critical lures.	• More Remember responses to items. corresponding to lists that were tested than from lists that were followed by arithmetic.<br>• No difference in proportion of remember responses for	Serial position data: strong primacy and recency effect; this inflates the overall probability of recall of studied items.

			from the study phase, 16 critical non-presented items, and 128 non-presented distractor items).<br>Participants also provided remember or know judgements for items they judged as “old”. Half the participants completed the recognition test 2 minutes after study.<br>*Test 2*<br>Half the participants completed the recognition test 1 day later.		• The decrease across delay was greater for lists followed by arithmetic than lists followed by recall.<br>• False recognition: critical lures > targets.	targets and critical lures following lists with recall. For lists with arithmetic, there were more remember judgements for critical lures than for targets.<br>*Test 2*<br>• Remember responses: immediate > 1 day.<br>• Remember judgements decreased across delay for lists followed by arithmetic but not lists followed by recall.	
Payne, Elie, Blackwell, & Neuschatz (1996), Experiment 2	*N* = 40	2(Recall Type: free recall, forced recall) x 3(Delay: immediate, 7 minutes, 14 minutes), mixed design, with Delay as the between-subjects factor.	*Study phase:*<br>Participants viewed 6, 10-word lists.<br>Presentation rate = 4 seconds/ word.<br>*Test 1:*<br>Half the participants completed 7 minutes of free recall and the other half completed 7 minutes of forced recall. During forced recall, participants were told they had to try and recall 60 words.<br>*Test 2*<br>This was repeated for T2, with 7 minutes of free or forced recall.<br>*Test 3*<br>This was repeated for T3, with 7 minutes of free or	• Critical lures: forced recall > free recall.<br>• Targets: free recall = forced recall.<br>• Critical lures increased across tests.<br>• Targets did not increase across tests.			

			forced recall. Following the recall tests participants went back and rated their confidence for each word that was presented at study.				
**2 Day Delay**	***N***	**Design**	**Method**	**Recall**	**Recognition**	**Remember Know**	**Other**
Bays, Foley, Madlener, & Haorei (2019)	*N* = 96	2(Delay: immediate, 2 days) x 3 (Encoding: solo, unfamiliar partner, familiar partner) mixed-design, with Encoding as the between-subjects factor.	*Study phase:*<br>Participants were randomly assigned to three conditions: Unfamiliar Partner (16 pairs), Familiar Partner (16 pairs), and Solo conditions. Participants read 10, 8-word lists (4 items presented from 2 thematic lists) and were told to create descriptions of things that the items could be used for.<br>*Test 1:*<br>7 minutes after the study phase, Participants completed an 80-item yes/no recognition test. For the pair conditions, participants were asked whether each item was assigned to them during the first phase of the experiment (Self), assigned to their partner (Partner), or a new item (New).<br>*Test 2:*<br>2 days after the study phase.		• Targets: immediate > 2 days.<br>• Targets decreased less in the Familiar Partner condition than the Unfamiliar Partner and Solo condition.<br>• Critical lures: 2 days > immediate.<br>• Critical lures: Familiar Partner and Unfamiliar Partner > Solo.<br>• Discriminability of targets and lures: immediate > 2 days.<br>• Discrimination: solo condition > familiar or unfamiliar partner conditions.		
Lampinen & Schwartz (2000), Experiment 1	*N* = 84	2(Delay: immediate, 2 days) x 2(Item Type: target, critical lure) mixed design, with Delay as the between-subjects factor.	*Study phase:*<br>12 auditory, 6-word lists, with half the lists presented in the study phase and half used as distractors for the recognition test. The recognition test consisted of 48 items, including 18 targets, 6 critical lures, 18 distractors and 6 distractor lures.<br>Presentation rate = 2 seconds/ word.<br>*Test 1:*		*Test 1*<br>• Corrected recognition (immediate testing): critical lures > targets.<br>*Test 2*<br>• Uncorrected recognition of targets < critical lures. However, corrected recognition showed that scores were lower over a 2-day delay than immediate testing.		The authors analyzed the data for uncorrected and corrected recognition. Corrected recognition accounts for response bias. Formulas for corrected targets and critical lures

			Half the participants immediately completed a recognition test; half completed a filler task.<br>*Test 2:*<br>2 days later, the remaining participants completed the recognition test. Participants from T1 who previously completed the recognition test performed a filler task.		• No interaction between delay and item type.		are listed below:<br>• Corrected target = (target – distractor)/(1 -distractor).<br>• Corrected critical lure = (Critical lure – distractor lure)/ (1-distractor lure).
Lampinen & Schwartz (2000), Experiment 2	*N* = 39	2(Delay: immediate, 2 days) x 2(Item Type: target, critical lure) mixed design, with Delay as the between-subjects factor.	The procedure was identical to Exp. 1, except participants made remember/know judgements for items they recognized.			*Test 1*<br>• Uncorrected and corrected recognition: critical lures > targets.<br>*Test 2*<br>• Uncorrected recognition: immediate > 2 days.<br>• Remember judgements for critical lures: Day 1 > Day 2. No interaction between Item Type and Delay.<br>• Corrected recognition was lower over 2 days. No interaction between Item Type and Delay.<br>• Uncorrected and corrected recognition (2 days): critical lures > targets.	Like Exp. 1, authors analyzed the data for uncorrected and corrected recognition.

Lampinen, Meier, Arnal, & Leding, (2005), Experiment 2	*N* = 40	2(List: presented, not presented) x 3 (Item Type: target, critical lure, missing exemplar) x 2 (Delay: immediate, 2 days) mixed design, with Delay as the between-subjects factor.	*Study phase:*<br>Participants viewed 8, 10-item lists. Participants performed a think-aloud task for each word, where they rated the pleasantness which served as a starting point to think about each word. Presentation rate = not reported.<br>*Test 1:*<br>Immediate recognition. A 64-item old/new task (16 targets, 8 critical lures, 8 missing associates from studied lists and 16 targets controls, 8 critical lures controls, 8 missing associate controls from unstudied lists). Participants provided remember/know judgements for old words. Participants "thought aloud" and said everything they were thinking about while making their judgements.<br>*Test 2:*<br>2 days later.		*Test 1:*<br>• Targets > Critical lures > Missing associates.<br>*Test 2:*<br>• Targets > Critical lures > Missing associates.<br>• Item Type x Delay interaction: fewer targets recognized at T2 but more of every other item type was recognized at T2.	*Test 1:*<br>• Remember judgements: Targets > Critical lures > Missing associates.<br>*Test 2:*<br>• Targets > Critical lures > Missing associates.<br>• Item Type x Delay interaction: fewer Targets were remembered at T2 but more of every other item type was remembered at T2.	*Test 1:*<br>• For all items except Targets and Critical lures, the distinctiveness of the word was the most common reason to reject an item at test.<br>• For Targets and Critical lures recollection rejection (remembering a different item was at study) was the most common reason to reject an item at test.<br>*Test 2:*<br>• Recollection rejections and distinctiveness declined across delay.<br>• For all items except Targets and Critical lures, the distinctiveness of the word was the most common reason to

							reject an item at test.<br>• Recollection rejection > distinctiveness for Critical lures and recollection judgement.
Price & Phenix (2015), Experiment 2	*N* = 110	2(Instructions: integration, no integration) x 2 (Delay: 1 day, 2 days) x 2 (Age: 6–9 years, 10–15 years) x 5(Item Type: RP+ [practiced], RP- [items from the practiced word list that were not practiced], Nrp [baseline list words that were not practiced], RP-^{L} [lure from a practiced list], Nrp^{L} [lure from an unpracticed list]) mixed design, with Instructions and Delay as between-subjects factors.	*Study phase:*<br>Children read 4, 14-word lists.<br>Presentation rate = 1.5 seconds/ word.<br>Half were told that each list of words ‘‘went together’’ and they should pay attention to how the words on the list were related (integration condition).<br>*Test 1:*<br>Following a 1-day delay, recall tasks took place. For the word stem task children were presented with a retrieval practice task in which half the words on two of the four lists were practiced (12 words in total). Word stems were presented. Children were asked to recall all the words they could from any of the lists. The first words from the lists were presented.<br>*Test 2:*<br>Following a 2-day Delay, participants completed the same word-stem task and the cued recall task.	• Older children reported more words than younger children.<br>• Retrieval-Induced Forgetting (RIF) in lures: older children > younger children.<br>• False recall reduced when children were given integration instructions before recall.<br>*Test 1:*<br>• RIF effect for the 1-day Delay, but not the 2-day Delay.<br>• In the 1-day Delay, RIF was observed in the no-integration condition and was marginal in the integration condition.<br>Test 2:<br>• No RIF effect for the 2-day Delay.<br>• In the 2-day delay, RIF was observed only in the no-integration condition and was not observed in the integration condition.			
**< 2-week Delay**			**Method**	**Recall**	**Recognition**	**Remember Know**	**Other**
Brainerd, Payne, Wright, Reyna (2003), Experiment 2	*N* = 80	2(Delay: immediate, 1 week) x 2 (Repetition: 1,	*Study phase:*<br>Participants heard 6, 15-word lists (3 presented once;	*Test 1*<br>• Targets < critical lures for items presented once.			

		3) x 2(Item Type: target, critical lure) x 3(Memory Test: 1, 2, 3) x 2(Time of Recall: 1st minute, 2nd minute) mixed design, with Delay as the between-subjects factor.	3 presented thrice); 2 minutes for free recall. Presentation rate = not reported.<br>*Test 1:*<br>Half the participants did 3 recall tests in succession right after hearing all 90 words on list. Each recall test was split by 2-minute buffers.<br>*Test 2:*<br>Same as Test 1, except participants completed testing 1 week later.	• Targets > critical lures for items presented thrice.<br>*Test 2*<br>• Targets < critical lures for items presented once.<br>• Targets = critical lures for items presented thrice.<br>• True recall declined over 1 week, while false recall remained stable.<br>• Slower recall, where the tendency for recall to occur during the first minute was more unlikely.			
Brainerd, Wright, Reyna, & Mojardin (2001), Experiment 2	*N* = 135	3(Instructions for Recognition: accept only Targets (V), accept only Related distractors (M), accept Targets or related distractors (VM) x 4 (Item: target, critical lure, list concept, missing associate (15th item)) x 2 (Associative Order: forwards, backwards) x 2(Delay: 2 minutes, 1 week) mixed design, with Instructions for Recognition as the between-subjects factor.	*Study Phase:*<br>Participants heard 16, 14-item lists, The lists were presented in a forward (8 lists) or backward (8 lists) order.<br>Presentation rate = 3 seconds/ word.<br>*Test 1:*<br>After each list, participants either recalled as many words as possible or completed a filler task – both activities lasted 90 seconds. Within the groups of forward lists and backward lists, participants completed a free recall test after 4 lists and a filler task after 4 lists.<br>*Test 2*:<br>Participants heard a 48-item test list, responding yes/no to each probe. The 8 lists included 4 forward lists (2 recall, 2 filler tasks) and 4 backward lists (2 recall, 2 filler tasks). List consisted of 4 groups of items: 24 targets, 8 critical distractors, 8 missing exemplars (unpresented 15th word for			Instructions for (V) was to accept targets. For the current paper this was interpreted as the standard DRM-instruction condition.<br><br>*A' Values*<br><br>*Test 1:*<br>Targets (V): .83<br>Targets (M): .47<br>Targets (VM): .85<br><br>Critical lures (V): .90<br>Critical lures (M): .39<br>Critical lures (VM): .91<br><br>*Test 2:*<br>Targets (V): .75<br>Targets (M): .60<br>Targets (VM): .75<br><br>Critical lures (V): .83	Note. We have collapsed means (i.e., forward presentation, backward presentation, recall, math) across additional conditions for the purpose of this table.

			each list), and 8 unrelated distractors.<br>*Test 3:*<br>1 week after study phase, participants completed a 96-item yes/no test. Half the items were the same as the items on T1 (48 items). The other test items were new (48 items: 24 targets, 8 critical distractors, 8 missing exemplars (unpresented 15th word of each list), and 8 unrelated distractors.			Critical lures (M): .51<br>Critical lures (VM): .82	
Cody, Steinman, & Teachman (2015)	*N* = 77	2(Delay: immediate, 3 days) x 2(Content: social, nonsocial) x 2 (Stressor Anticipation Group: present, absent) x 2 (Social Anxiety Group: SAD, Control) mixed design, with Stressor Anticipation Group and Social Anxiety Group as the between-subject factors.	*Study phase:*<br>Participants rated their baseline anxiety on the Subjective Units of Distress Scale (SUDS). Stressor present participants learned they would later give a speech in front of the experimenter. Stressor absent participants learned they would later complete questionnaires. Participants repeated the SUDS. Participants then viewed 12, 12-word lists consisting of social and nonsocial words. Presentation rate = 1.75 seconds/ word. After the first block of words were presented, participants completed a 3-minute distractor, followed by an inclusion recall task. Participants recalled words and related words from the first block and noted the related words. Participants were given 90 seconds to record their answers for each block of words for the inclusion recall task. After 90 seconds of recall, the	• Words recalled: Immediate (T1) > 3 days (T2)<br>• SAD group produced more non-social critical lures than the Control group did in the Stressor Present condition.<br>*Test 1*<br>• Recall for targets and critical lures was positively associated at both testing sessions.<br>• SUDS was positively correlated with recall of social critical lures at T2.<br>• Stressor Absent Group: SUDS was correlated with non-social critical lures at T1 and with social and non-social critical lures at T2.<br>• True recall: More nonsocial words were recalled than social words.<br>*Test 2*<br>• Stressor Absent group: there were fewer critical lures during T2 than in T1.<br>• Stressor Absent Group: at T2, SUDS was correlated with non-social critical lures at T2.<br>• Recall of critical lures was persistent across T1 and T2			

			second block of items appeared. Participants completed another distractor task and then repeated the inclusion recall task for the second block.<br>*Test phase:*<br>3 days later, participants were reminded of their task associated with the stressor condition and completed the SUDS, before recalling as many words as possible from the study phase, using the inclusion recall task. Then, both groups gave a brief videotaped speech (up to 5 minutes).	for the Stressor Present participants.			
Howe, Candel, Otgaar, Malone, & Wimmer (2010), Experiment 3	*N* = 60	2(Item: true, false) x 2(Valence: neutral, negative) x 2(Delay: immediate, 1 week) mixed design, with Delay as the between-subjects factor.	*Study phase:*<br>12, 10-item DRM lists were presented, 6 of which were negative lists and 6 were neutral lists.<br>Presentation rate = 3 seconds/ word.<br>Once participants heard a word list, they completed a 30-second filler and then the recall task. This procedure was repeated until all 12 lists were presented.<br>*Test 1:*<br>Immediately after the final list, half the participants completed a 72-item yes/no recognition test. The recognition test consisted of 36 targets, 12 critical lures, 12 non-presented semantically related words and 12 non-presented unrelated words.<br>*Test 2:*<br>1 week later, the remaining half were given the same recognition test as T1.		*Test 1*<br>• True recognition > false recognition.<br>• Compared to neutral critical lures, false recognition of critical lures was higher for negative lists in the immediate testing condition.<br>*Test 2*<br>• True recognition decreased for targets from neutral lists.<br>• Critical lures from neutral lists remained stable.<br>• True recognition of targets decreased for negative lists.<br>• Critical lures from negative lists increased.		In this series of experiments, we are only interested in the dependent variables that manipulate delay (i.e., recognition).

Howe, Candel, Otgaar, Malone, & Wimmer (2010), Experiment 4	*N* = 30	2(Age: 5-year-olds, 8-year-olds) x 2(Delay: immediate, 1 week) x 2(Item: true, false) x 2(Valence: neutral, negative) mixed design, with Age and Delay as the between-subject factors.	The procedure resembled Exp. 3, except:<br>• The sample consisted of 5 and 8-year-old children.<br>• 8, 10-item DRM lists were presented, 4 of which were negative lists and 4 were neutral lists.<br>• The 48-item recognition test consisted of 24 targets, 8 critical lures, 8 semantically related distractors, 4 unrelated neutral distractors and 4 unrelated negative distractors.		*Test 1*<br>• 5-year-olds < 8-year-olds.<br>• Valence had no effect on immediate recognition of critical lures.<br>*Test 2*<br>• True recognition of targets decreased but false recognition of critical lures remained stable for neutral lists and increased for negative lists.<br>• For neutral lists, true recognition of targets decreased over a 1-week delay. There was no change in false recognition of critical lures.<br>• For negative lists, true recognition of targets decreased, while false recognition of critical lures increased over a 1-week delay. These effects did not vary with age.		
Howe, Candel, Otgaar, Malone, & Wimmer (2010), Experiment 5	*N* = 80	2(Age: 7- year-olds, 11-year-olds) x 2(Delay: immediate, 1 week) x 2(Item: true, false) x 2(Valence: neutral, negative) mixed design, with Age and Delay as the between-subject factors.	The procedure resembled Exp. 3, except:<br>• The sample consisted of 7- and 11-year-olds.<br>• 10, 10-item lists were presented, 5 of which were negative lists and 5 were neutral lists. 2 additional items were used as distractors in the recognition test.<br>• The 78-item recognition test consisted of 40 targets, 10 critical lures, 10 semantically related distractors, 12 unrelated		*Test 1*<br>• 7- year-olds < 11-year-olds.<br>• True recognition of targets was higher than false recognition of critical lures in the immediate-testing condition.<br>*Test 2*<br>• False recognition of critical lures was higher than true recognition of targets over a 1-week delay.		

			neutral distractors (6 taken from unstudied lists) and 6 unrelated negative distractors.		• True recognition of targets decreased for neutral and negative items at similar rates over a 1-week delay.<br>• False recognition of critical lures for neutral items was stable over a 1-week delay, while false recognition of critical lures for negative items increased.		
Knott & Shah (2019)	*N* = 68	2(Presentation Speed: 20 milliseconds, 2 seconds) x 2(List Valence: Neutral, Negative) x 2(Delay: immediate, 1 week) mixed design, with Delay as the between-subjects factor.	*Study phase:*<br>20, 12-words lists were presented, with 10 negative and 10 neutral lists. The recognition test had 120 words: 20 critical lures, 60 list items, 20 weak-related fillers and 20 unrelated fillers. There were 2 main blocks of lists that were presented visually. Participants either read 10 negative lists in the first block followed by 10 neutral lists in the second block, or the reverse. Each block was split into 2 additional groups based on presentation rate:<br>Presentation rate group one: 20 milliseconds/ list (five lists).<br>Presentation rate group two: 2 seconds/ list (five lists).<br>Each group was followed by a 1-minute break. There was a 5-minute break between blocks that included a filler task.<br>*Test 1:*<br>Half the participants completed an immediate recognition test (following another 5-minute filler).		*Test 1*<br>• Targets: 2 seconds > 20 milliseconds.<br>• There was a higher rate of correct recognition in the negative condition compared to the neutral condition.<br>• Critical lures, neutral lures = negative lures.<br>*Test 2*<br>• False responses to critical lures: 2 seconds > 20 milliseconds.<br>• Targets for lists presented at a 20-millisecond rate increased over 1 week.<br>• Critical lures: immediate < 1 week<br>• Critical lures (neutral lures): immediate = 1 week.<br>• Critical lures (negative lures): 1 week > immediate.<br>• False memories: negative critical lures > neutral critical lures.<br>• False recognition for weak fillers: 1 week > immediate.		

			Participants made an old/new recognition, followed by a recollective experience judgement of remember/know/guess (only if an old response was made).<br>*Test 2:*<br>1 week later, the second half completed the same recognition test.		• False recognition for old responses to unrelated fillers: 1 week > immediate.<br>• Increase in false responses after 1 week only significant for negative distractors, not neutral distractors.		
Knott & Thorley, (2014)	*N* =-48	2(Mood: neutral, negative) x 2(Stimuli: neutral, negative) x 2(Delay: immediate, 1 week) mixed design, with Mood and Delay as the between-subject factors.	*Study phase:*<br>Participants completed a valence measure and watched either negative or neutral 5-minute video clips. This was followed by the same valence measure, and then an additional, new neutral video to induce neutral mood. After, they heard 12, 12-word. Presentation rate = 3 seconds/ word. Half the lists were negative, while the other half were neutral. Participants then completed the valence measure.<br>*Test 1:*<br>Participants received an immediate recognition test, which consisted of half the lists that were studied at encoding. This test consisted of 6 critical lures (3 negative, 3 neutral), 24 targets (2 items from 6 negative lists, 2 items from 6 neutral lists) and 18 unrelated and non-studied fillers (9 negative, 9 neutral). Each item required an old/new response and remember/know/guess judgements. If an item was labeled old, participants had		*Test 1*<br>• Critical lures (old responses): negative lures = neutral lures.<br>• No difference in targets between stimuli for old responses.<br>• No effects of mood for false recognition of old responses.<br>*Test 2*<br>• Critical lures (old responses): negative lures > neutral lures.<br>• Targets: negative stimuli > neutral stimuli.<br>• Targets and critical lures for guess responses were low, but targets increased over time.	*Test 1*<br>• Targets (remember responses): neutral stimuli > negative stimuli.<br>• No difference in critical lures for neutral lures (remember responses).<br>• Critical lures (know responses): negative lures = neutral lures.<br>*Test 2*<br>• Decrease in targets for remember responses.<br>• Targets (remember responses): negative stimuli > neutral stimuli.<br>• Critical lures (remember responses): negative lures > neutral lures.<br>• Critical lures (remember responses): no decrease in	Recognition test responses (i.e., old, remember, know, guess) were analyzed separately.

			to make additional remember/know/guess judgements.<br>*Test 2:*<br>Participants returned 1 week later to complete another recognition test consisting of the second half of the lists that were studied at encoding. This test consisted of critical lures, remaining studied targets and an additional 18 unrelated fillers. The same recognition test and remember/know/guess were administered.			negative lures, but neutral lures decreased.<br>• No difference in critical lures for neutral lures (remember responses).<br>• Critical lures (know responses): negative lures > neutral lures.	
Packard, Rodríguez-Fornells, Stein, Nicolás, & Fuentemilla (2014), Experiment 1	N = 46	2(Condition: fearful, neutral) x 2(Delay: immediate, 2 weeks) x 2(Item Type: verbatim, gist-based), mixed design with Delay as the between-subjects factor.	*Study phase:*<br>Participants heard 14, 12-word lists.<br>Presentation rate = 15 seconds/ list. Words were encoded under neutral contexts (neutral context) or under anticipation of electric shocks (fearful context).<br>For each list there were 6 test items: 3 words presented from the study phase, 2 were unrelated words, and 1 was the critical lure.<br>*Test 1:*<br>30 minutes after study phase. After each item presentation, an 'old/new' discrimination was required. 'Old' choice on recognition test was followed by a Remember/Know judgment. If participants chose 'New,' they were asked whether they were 'Sure' or 'Not-Sure.'<br>*Test 2:*		• Encoding was higher under fearful than under neutral contexts.<br>*Test 1*<br>• Verbatim memory was compromised when encoded under the fearful context condition during immediate testing condition.<br>*Test 2*<br>• Verbatim words showed a main effect of fearful condition and a main effect of delay.<br>• Decrease in verbatim memory due to fearful context condition during immediate testing condition but not delay testing condition.<br>• Gist-based memories were unaffected in both immediate testing condition and delay testing condition.		

			6–8 days later. T2 procedures were identical to T1.		• Fearful context-dependent effect on verbatim memories not observed after 1 week.		
Packard, Rodríguez-Fornells, Stein, Nicolás, & Fuentemilla (2014), Experiment 2	*N* = 39	2(Condition: fearful, neutral) x 2(Delay: immediate, 2 weeks) x 2(Item Type: verbatim, gist-based), mixed design, with Delay as the between-subjects factor.	Delay was increased to 2 weeks<br>Exp. 2 followed the same procedure as Exp. 1, except after the recognition test, words were presented again, and participants were asked if the words were presented in the fearful or neutral context.		• Explicit encoding context memory was marginally higher for critical lure items > targets.<br>• Verbatim items showed a marginal effect of fearful condition and a main effect of delay.		
Seamon, Luo, Schlegel, Greene, & Goldenberg (2000), Experiment 1	*N* = 120	3(Age: 6-7-year-olds, 10-12-year-olds, 17-22-year-olds) x 2(Delay: immediate, 3 days) x 2(Stimulus Set: A, B) between-subjects design.	*Study phase:*<br>Participants viewed 6 sets of 9 categorically related pictures.<br>Presentation rate = self-paced with 2-second inter-stimuli interval.<br>*Test 1*<br>Half of the participants in each group completed an immediate yes/no recognition test.<br>*Test 2*<br>3-days later, the remaining participants did a 60-item yes/no picture recognition test (18 targets and 12 critical lures from studied picture sets and 18 targets and 12 critical lures from unstudied picture sets.		• T1 targets > T2 targets.<br>• T1 critical lures < T2 critical lures.<br>• A’ for Target memory T1 > A’ for Target memory T2.<br>• A’ of T1 critical lures < A’ for T2 of critical lures.<br>*Test 1*<br>• No effect of Age.<br>*Test 2*<br>• A’ for Targets, 6-7 years < 10-12 years and adults.<br>• No effect of Age for A’ of Critical lure recognition.<br>• Falsely recognized related critical lures > unrelated critical lures on immediate and delayed testing.		
Sherman, Follows, Mushore, Hampson-Jones & Wright-Bevans (2015), Experiment 2	*N* = 40	2(Presentation Format: blocked, random) x 2(Delay: immediate, 1 week) x 3(Stimulus	*Study phase:*<br>3 categories (banks, cars, beers) were chosen for lists. 5 adverts for each category were chosen and most popular advert was the critical lure for the lists.<br>Participants watched a			*Test 2*<br>• More items recognized at T2 than at T1 (.61 vs. .49).<br>• False recognition of	Presentation length per word was not reported for this experiment.

		Type: list item, lure, unrelated items) mixed design, with Presentation Format and Delay as the between-subject factors.	recording of a television show and viewed advertisements.<br>*Test 1:*<br>Half the participants were asked to complete a remember/know/guess recognition test for the brands seen during the advert breaks. The recognition test consisted of 3 presented brands, 3 related, unpresented lure brands and 6 unrelated brands.<br>*Test 2*<br>The other half returned 1-week later to complete the recognition test.			lures increased over time (.47 vs. .85).<br>• More remember responses at T2 than at T1 (.38 vs .27).<br>• Remember responses to lures increased over time (.13 vs. .42).<br>• False recognition increased over time.	
Sherman & Kennerley (2014), Experiment 1A	*N* = 32	2(Stimulus: list item, lure) x 2(Delay: immediate, 1 week) within-subjects design.	*Study phase:*<br>9 musical artists were chosen, and their most popular song was used as the critical non-presented lure, while the 5 next most popular songs made up the list to be presented at study. Presentation rate = 2.5 seconds/ song title.<br>*Test 1:*<br>There was a 5-minute distractor task, followed by free recall.<br>*Test 2:*<br>Participants completed another free recall test 1-week later.	*Test 1*<br>• 14 items recalled on average.<br>• More items recalled immediately than 1-week later (17% vs 13%).<br>*Test 2*<br>• 9 items recalled on average.<br>• Target recall decreased over 1 week (28% vs 14%).<br>• Critical lure recall increased over 1 week (5% vs 12%).			
Sherman & Kennerley (2014), Experiment 1B	*N* = 35	2(Stimulus: list item, lure) x 2(Delay: immediate, 1 week) within-subjects design.	The same artists and song titles were used as for Exp. 1A.<br>Presentation rate = 30 seconds/ song clip.<br>Each of the 5 songs was presented for each artist were recorded to form lists.<br>The procedure was the same as Exp. 1A, except that	*Test 1*<br>• 19 items recalled on average.<br>• More items recalled immediately than 1 week later (27% vs 23%).<br>• More items recalled immediately than 1 week later in both conditions (22% vs 18%).			

			instead of watching song names, participants heard the song clips.	*Test 2*<br>• 11 items recalled on average.<br>• Target recall decreased over 1 week (34% vs 14%).<br>• Critical lure recall increased over 1 week (21% vs 33%).<br>• More items recalled in the song clips condition than the song titles condition (25% vs 15%).<br>• More list items were recalled compared to lures in both conditions (22% vs 18%).			
Thapar & McDermott (2001), Experiment 1	*N* = 99	3(Encoding Task: color, vowels, pleasantness) x 3(Delay: immediate, 2 days, 7 days) mixed design, with Delay as the between-subjects factor.	*Study phase:*<br>Participants viewed 24, 12-item lists.<br>Presentation rate = 4 seconds/ word. They were asked to encode them based on one of three rules: 1) rate the pleasantness 2) count the vowels 3) write the colour of the word.<br>*Test 1:*<br>The first group of participants were asked to write down as many words as they could recall immediately.<br>*Test 2*<br>2 days later, the second group of participants were asked to write down as many words as they could recall.<br>*Test 3*<br>7 days later, the third group of participants were asked to write down as many words as they could recall.	• Decline across delay for targets processed deeply (pleasantness condition) and shallowly (color condition; vowel condition).<br>• A decline across delay for critical non-presented items occurred in all three encoding conditions.<br>• Targets and critical lures were greater in the deep processing (pleasantness) condition.<br>*Test 2*<br>• Targets and critical lures: immediate > 2-day delay.<br>• Targets: 2 day > 7 days (only marginally significant).<br>• The decrease was greater for targets than critical lures.<br>• The decrease in targets and critical lures was steeper in the pleasantness condition. This may have been due to floor effects in the shallow processing conditions.<br>*Test 3:*<br>• After 1 week, probability of recalling targets and critical			

				lures reversed. T1: targets (.18) and critical lures (.12); after 1 week: targets (.05) and critical lures (.07).			
Thapar & McDermott (2001), Experiment 2	*N* = 72	3(Encoding Task: color, vowels, pleasantness) x 3(Delay: immediate, 2 days, 7 days) mixed design with Delay as the between-subjects factor.	*Study phase:*<br>The procedure for this phase was the same as Exp. 1, except that that a yes/no recognition test replaced the free recall test used in Exp. 1.<br>*Test 1:*<br>The first group of participants completed a 144-item recognition test. The recognition test included 24 words presented from the study phase, 24 critical non-presented targets, and 24 weakly related non-presented words.<br>*Test 2*<br>2 days later, the second group of participants completed the same recognition test from T1.<br>*Test 3*<br>7 days later, the third group of participants completed the same recognition test from T1.		*Test 1*<br>• Targets > critical lures.<br>*Test 2*<br>• Targets = critical lures.<br>• Targets and critical lures decreased from immediate to the 2-day Delay.<br>• Steeper decrease in recognition for targets than critical lures.<br>*Test 3*<br>• Targets for Encoding (pleasantness): immediate > 2-day or 7-day Delay.<br>• Critical lures for Encoding (pleasantness): immediate > 2-day or 7-day Delay.<br>• Deep processing (pleasantness) led to greater decline across delay than shallow processing.		
Wang, Otgaar, Howe, Smeets, Merckelbach, & Nahouli (2017), Experiment 2	*N* = 71	2(Delay: immediate, 1 week) × 2(Memory Type: critical lures, studied items) × 2(Belief: challenged, control) mixed design, with Delay as the between-subjects factor.	*Study phase:*<br>Participants viewed 24 lists with 10 associates. Presentation rate = 1.5 seconds/ word.<br>*Test 1:*<br>After a 3-minute filler, participants completed an immediate 96-item recognition test: 12 non-presented critical lures, 48 studied items, and 36 unrelated items. Participants were told that previous words they recognized were		• No effects of Delay.		Items that were challenged in a way that undermined the participants' beliefs resulted in fewer CRAT solutions. This applied to targets and critical lures and did not vary across delay. Therefore,

			wrong, or they were told that they got previous words correct.<br>*Test 2:*<br>Either immediately following the recognition test or 1 week later, participants completed a 24-item compound remote associate task (CRAT), (12 critical lures and 12 targets).				challenging the beliefs about critical lures influences the ability of false memories to prime CRATs. This effect was also seen on target memories.
**> 2-week Delay**		**Design**	**Method**	**Recall**	**Recognition**	**Remember Know**	**Other**
Brainerd & Reyna (1996), Experiment 1	*N* = 80	2(Age: 5-year-olds, 8-year-olds) x 2(Degree of Learning: high, low) x 2(Prior Testing Status: tested, untested) x 2(Delay: immediate, 1 week) mixed design, with Age as the between-subjects factor.	*Study phase:*<br>Children heard 100 words. Presentation rate = 3 seconds/ word.<br>There were 40 critical targets (related), and 60 non-critical targets. Half the items were read once (Degree of Learning: high). The other half were read thrice (Degree of Learning: low).<br>*Test 1:*<br>Following a 5-minute buffer, participants completed a 60-item yes/no recognition test: 30 distractors and 30 targets (half were old and half were new).<br>*Test 2*<br>1 week later, participants completed a 120-item test: 30 new distractors, 30 new targets, and 60 items from T1.		• Targets: .93 (high learning) and .68 (low learning) for 5-year-olds<br>• Targets: .95 (high learning) and .75 (low learning) for 8-year-olds.<br>*Test 2*<br>• Targets: previously tested > previously untested for all age groups.<br>• Critical lures on immediate test preserved at 1-week delay.<br>• 5- and 8-year-olds falsely accepted category names and same-category exemplars at higher rates if those distractors had been previously tested than if they had not been.<br>• Targets: 5-year-olds < 8-year-olds.<br>• Immediate test increased false acceptance rates on the delayed test.<br>• Immediate testing did not increase acceptance		• *d’* values (true – false memory) values were also computed for each participant for all learning combinations, prior testing and types of distractors. Children could distinguish between targets presented during the study phase from distractors at 1-week delayed testing.<br>• 5-year-olds: false-memory > true-memory at immediate testing. This finding was

					for unrelated distractors.<br>• Distractors that were falsely accepted at T1 were then falsely accepted at T2, with previously rejected distractors later falsely accepted.		not significant for 8-year-olds.
Brainerd & Reyna (1996), Experiment 2	*N* = 80	2(Age: 5-year-olds, 8-year-olds) x 2(Degree of Learning: high, low) x 2(Prior Testing Status: tested, untested) x 2(Delay: immediate, 1 week, 2 weeks) mixed design, with Age as the between-subjects factor.	Same as Exp. 1, except for the following changes:<br>*Test 1*<br>Immediate testing.<br>*Test 2*<br>1-week delayed testing.<br>*Test 3*<br>1-week delayed testing after T2.		• False memory creation effect for 5-year-olds: gist-sharing distractors > unrelated distractors.<br>*Test 1*<br>• Overall targets: .54 for 5-year-olds and .59 for 8-year-olds.<br>*Test 2*<br>• T1 test increased critical lures for meaning-sharing distractors at T2.<br>*Test 3*<br>• Targets: 8-year-olds > 5-year-olds.<br>• Targets: high Degree of Learning > low Degree of Learning.<br>• Critical lures: tested distractors > untested distractors for 5-year-olds but not 8-year-olds.		• *d'* values (true – false memory) values were also computed for each participant for all learning combinations, prior testing and types of distractors.
Brainerd & Reyna (2018), Experiment 3	*N* = 168	2(Status: old-similar judgement, new-similar judgment) x 3(Item: strong new-similar, weak new-similar, old-similar) within-subjects design.	*Study phase:*<br>Participants heard 16, 8-word lists.<br>Presentation rate = 3 seconds/ word.<br>*Test 1:*<br>This was followed by an immediate 72-item recognition test, which consisted of 8 lists of 24 Old words (3 from each of the 8 presented lists), 8 strong critical lures, 16 weak lures		*Test 1*<br>• When asked p(Old?), Participants can't distinguish Old items from critical lures.<br>• When asked p(NS?), participants can distinguish Old from critical lures: p(NS?) is greater for critical lures than for Old.<br>• When asked p(Old?), Participants can		Delay was not compared statistically. All values are bias-corrected expressions of saying, "yes" when asked Old? Or when asked New Similar [critical lure]?

			(2 unpresented words from each of the 8 lists drawn randomly from positions 9-15), and 24 New words (8 critical distractors from 8 unpresented lists, plus 2 words from each of those lists).<br>*Test 2:*<br>Participants returned 7-14 days later (*avg* = 10 days) and completed 144-item recognition test, including 72 words from T1 plus 72 words from the 8 lists not tested at T1. Participants learned that test contained list words (Old), new words that were semantically similar to list words (NS), and new words that were unrelated to list words (New); Participants judged Old/New for ½ the words; Participants judged New-Similar for half the words [thus, 2 DVs].		distinguish Old from critical lures; when asked p(NS?), Participants can distinguish Old from critical lures.<br>*Test 2*<br>• For same items tested on immediate test, when asked p(Old?), participants can distinguish Old from critical lures.<br>• When asked p(NS?), Participants can distinguish Old from critical lures: p(NS?) is greater for critical lures than for Old.<br>• For different items (not tested on immediate test), when asked p(Old?), Participants can't distinguish Old from critical lures: when asked p(NS?), Participants can distinguish Old from critical lures.<br>**Take-home**: using standard task (probability Old) on same or different items in immediate and delayed test resulted in higher hits and false alarms on immediate than delayed test.		
Carneiro, Garcia-Marques, Lapa & Fernandez (2017), Experiment 1	*N* = 128	2(Type of Critical Item: associative critical items, thematic items) x 2(Delay: immediate, 1 week) x 2 (Status of	*Study phase:*<br>15, 10-word lists were presented, with 12 target lists and 3 filler lists. Lists were presented auditorily. Presentation rate = 2 seconds/ word.<br>For each list, two critical lures were used: the		Test 1:<br>• Targets > critical lures.<br>• Target immediate AIs = Target immediate Tis.<br>• Critical lures: immediate AIs > immediate TIs.<br>Test 2:		• Associative Critical Items (AI).<br>• Thematic Critical Items (TI).<br>• For the delay condition, the yes/no

		Critical Item: in lists [targets], out of lists [critical lures]) mixed design, with Delay and Status of Critical Item as the between-subject factors.	associative critical lures (AI) and the thematic critical lures (TI).<br>*Test 1:*<br>Immediate 4-alternative recognition test for 12 target lists. Memory for 3 filler lists was not tested. Recognition for target lists consisted of 108 pairs of words, where the AI, the TI, the first and fifth associate, and one unrelated/non-presented item was tested.<br>*Test 2:*<br>Participants performed the same yes/no recognition test immediately after the lists were presented and 1-week after, they performed a surprise 4-alternative recognition test.		• Targets: immediate > 1 week.<br>• Targets: immediate AIs and TIs > 1-week AIs and TIs.<br>• Targets: 1-week AIs > 1-week TIs.<br>• Critical lures: 1-week AIs = 1-week TIs.<br>• Critical lures: immediate = 1 week.<br>• Critical lures: AIs immediate > AIs 1-week.<br>• Critical lures: TIs immediate = TIs 1-week.<br>• Critical lures: AIs > TIs.		recognition test was excluded from the analyses, as its main purpose was to limit participants' expectations of a delayed test and prevent practice effects with the 4-alternative recognition test.
Carneiro, Garcia-Marques, Lapa, & Fernandez (2017), Experiment 2	*N* = 117	2(Type of Critical Item: associative critical items, thematic items) x 2(Delay: immediate, 1 week) x 2(Retrieval Time: self-paced, speeded) mixed design, with Delay and Retrieval Time as the between-subject factors.	*Study phase:*<br>The procedure was the same as Exp. 1, but only 10 pairs in each list were presented. In the speeded condition, participants responded within 0.8-seconds (0.4-seconds for each word presentation and 0.4-seconds for a response).<br>*Test 1:*<br>Immediate testing. Participants determined whether the item was old or new. For the delay condition, memory for the filler lists was tested immediately.<br>*Test 2:*<br>Memory for the 10 target lists was tested 1 week later. In both T1 and T2, the recognition test consisted of 68 words (20 words from the selection of 2 studied words		• Targets: self-paced > speeded.<br>• Critical lures: speeded > self-paced.<br>*Test 2*<br>• Targets: Immediate > 1 week.<br>• Critical lures: Immediate > 1 week.<br>• Difference between immediate and delay for AIs.<br>• No difference between immediate and delay for TIs.<br>• No difference in Delay for critical lures of TIs in self-paced condition.<br>• Critical lures of TIs in speeded condition: immediate > 1 week.<br>• For all items except TIs: immediate < 1 week.		• Associative Critical Items (AI).<br>• Thematic Critical Items (TI).

			per target list; 10 critical associative items of presented target lists; 10 critical thematic items of presented target lists; and 28 unrelated items). Filler test consisted of 30 items (5 old items from each of the 3 filler lists and 15 new words).		• Immediate condition: studied items were recognized more than unpresented Als. No difference between Type of critical lures over 1 week.<br>• False recognition for unrelated items: immediate < 1 week; speeded condition > self-paced condition.		
Seamon, Luo, Kopecky, Price, Rothschild, Fung, & Schwartz, (2002), Experiment 1	*N* = 60	Single factor (Delay: immediate, 2 weeks, 2 months) between-subjects design.	*Study Phase:*<br>Participants heard 8, 15-word lists.<br>Presentation rate = 1.5 seconds/ word.<br>*Test 1*<br>Immediate free recall<br>*Test 2*<br>2-week delayed free recall<br>*Test 3*<br>2-month delayed free recall	• Critical lures > Targets at each Test.<br>• Targets: T1 < T2 < T3. The decrease was greater from T1 to T2.<br>• Critical lures were stable from T1 to T2. False recall of critical lures decreased from T2 to T3.			
Seamon, Luo, Kopecky, Price, Rothschild, Fung, & Schwartz, (2002), Experiment 2	*N* = 120	Single factor (Delay: immediate, 2 days, 2 weeks, 2 months) between-subjects design.	*Study Phase:*<br>Participants heard 8, 15-word lists presented at 1.5 seconds per word.<br>*Test 1*<br>Immediate recognition.<br>*Test 2*<br>2-day delayed recognition.<br>*Test 3*<br>2-week delayed recognition.<br>*Test 4*<br>2-month delayed recognition.<br>Participants completed a 64-item yes/no recognition test (24 targets and 8 critical lures from studied lists, 24 targets and 8 critical lures from non-studied lists), along with remember/know judgements. The recognition test was similar across tests.		• Recognition in all delay conditions: targets > critical lures; related > unrelated critical items.<br>• Pr scores: critical lures > targets, except for at T4 where scores were about chance for both false recognition and target recognition.<br>• *d':* critical lures > targets.<br>• Targets and critical lures: Immediate > 2 days = 2 week > 2 months.<br>• *A'* Targets and critical lures: Immediate > 2 days = 2 weeks > 2 months.	*Test 1*<br>• Remember > Know judgements for Targets and Critical lures from studied lists.<br>• Remember < Know judgements for Targets and Critical lures from unstudied lists.<br>*Test 4*<br>• Remember = Know judgements for Targets and Critical lures from studied lists.	• Pr – a high-threshold measure.<br>• d'- signal detection measure.

						• Remember < Know judgements for Targets and Critical lures from unstudied lists.	
Toglia, Neuschatz, & Goodwin (1999), Experiment 2	*N* = 154	2(Presentation Format: blocked, random) x 3(Delay: immediate, 1 week, 3 weeks) between-subjects design.	*Study phase:* Participants heard 5, 12-word lists, which were presented either blocked by meaning or randomized. Presentation rate = not reported. *Test 1:* Participants immediately recalled as many words as possible for 5 minutes. *Test 2:* 1-week recall *Test 3:* 1-week recall after Test 2.	*Test 3* • Targets: immediate > 1 week and 3 weeks. • Targets: 1 week = 3 weeks. • Critical lures: immediate > 1 week < 3 weeks. This effect was large across Delay. • Critical lures: blocked > random. This was observed immediately and after 3 weeks, but not after 1 week.			Adjusted Ratio of Clustering (ARC): • Recall of targets: blocked > random. This was observed immediately, but not after 1 or 3 weeks. • Recall of critical lures: blocked > random. This was observed immediately and after 3 weeks. Confidence: • Recall of targets: immediate > 1 or 3 weeks. • Recall of critical lures: No differences in confidence ratings across Delay.

Note: N = 40. This table only reports results pertaining to delay. Exp. refers to Experiment. T1 refers to Time 1 and T2 to Time 2.
